# Genetic vulnerability and adverse mental health outcomes following mild traumatic brain injury: a meta-analysis of CENTER-TBI and TRACK-TBI cohorts

**DOI:** 10.1016/j.eclinm.2024.102956

**Published:** 2024-12-05

**Authors:** Mart Kals, Lindsay Wilson, Daniel F. Levey, Livia Parodi, Ewout W. Steyerberg, Sylvia Richardson, Feng He, Xiaoying Sun, Sonia Jain, Aarno Palotie, Samuli Ripatti, Jonathan Rosand, Geoff T. Manley, Andrew I.R. Maas, Murray B. Stein, David K. Menon, The Genetic Associations In Neurotrauma (GAIN) Consortium (with contribution from the CENTER-TBI and TRACK-TBI studies), Cecilia Ackerlund, Hadie Adams, Krisztina Amrein, Nada Andelic, Lasse Andreassen, Audny Anke, Anna Antoni, Gérard Audibert, Philippe Azouvi, Maria Luisa Azzolini, Ronald Bartels, Pál Barzó, Romuald Beauvais, Ronny Beer, Bo-Michael Bellander, Antonio Belli, Habib Benali, Maurizio Berardino, Luigi Beretta, Morten Blaabjerg, Peter Bragge, Alexandra Brazinova, Vibeke Brinck, Joanne Brooker, Camilla Brorsson, Andras Buki, Monika Bullinger, Manuel Cabeleira, Alessio Caccioppola, Emiliana Calappi, Maria Rosa Calvi, Peter Cameron, Guillermo Carbayo Lozano, Marco Carbonara, Ana M. Castaño-León, Simona Cavallo, Giorgio Chevallard, Arturo Chieregato, Giuseppe Citerio, Hans Clusmann, Mark Steven Coburn, Jonathan Coles, Jamie D. Cooper, Marta Correia, Amra Čović, Nicola Curry, Endre Czeiter, Marek Czosnyka, Claire Dahyot-Fizelier, Paul Dark, Helen Dawes, Véronique De Keyser, Vincent Degos, Francesco Della Corte, Hugo den Boogert, Bart Depreitere, Đula Đilvesi, Abhishek Dixit, Emma Donoghue, Jens Dreier, Guy-Loup Dulière, Ari Ercole, Patrick Esser, Erzsébet Ezer, Martin Fabricius, Valery L. Feigin, Kelly Foks, Shirin Frisvold, Alex Furmanov, Pablo Gagliardo, Damien Galanaud, Dashiell Gantner, Guoyi Gao, Pradeep George, Alexandre Ghuysen, Lelde Giga, Ben Glocker, Jagoš Golubović, Pedro A. Gomez, Johannes Gratz, Benjamin Gravesteijn, Francesca Grossi, Russell L. Gruen, Deepak Gupta, Juanita A. Haagsma, Iain Haitsma, Raimund Helbok, Eirik Helseth, Lindsay Horton, Jilske Huijben, Peter J. Hutchinson, Bram Jacobs, Stefan Jankowski, Mike Jarrett, Ji-yao Jiang, Faye Johnson, Kelly Jones, Mladen Karan, Angelos G. Kolias, Erwin Kompanje, Daniel Kondziella, Lars-Owe Koskinen, Noémi Kovács, Ana Kowark, Alfonso Lagares, Linda Lanyon, Steven Laureys, Fiona Lecky, Didier Ledoux, Rolf Lefering, Valerie Legrand, Aurelie Lejeune, Leon Levi, Roger Lightfoot, Hester Lingsma, Marc Maegele, Marek Majdan, Alex Manara, Hugues Maréchal, Costanza Martino, Julia Mattern, Charles McFadyen, Catherine McMahon, Béla Melegh, Tomas Menovsky, Ana Mikolic, Benoit Misset, Visakh Muraleedharan, Lynnette Murray, Ancuta Negru, David Nelson, Virginia Newcombe, Daan Nieboer, József Nyirádi, Matej Oresic, Fabrizio Ortolano, Olubukola Otesile, Paul M. Parizel, Jean-François Payen, Natascha Perera, Vincent Perlbarg, Paolo Persona, Wilco Peul, Anna Piippo-Karjalainen, Matti Pirinen, Dana Pisica, Horia Ples, Suzanne Polinder, Inigo Pomposo, Jussi P. Posti, Louis Puybasset, Andreea Rădoi, Arminas Ragauskas, Rahul Raj, Malinka Rambadagalla, Veronika Rehorčíková, Isabel Retel Helmrich, Jonathan Rhodes, Sophie Richter, Saulius Rocka, Cecilie Roe, Olav Roise, Jeffrey Rosenfeld, Christina Rosenlund, Guy Rosenthal, Rolf Rossaint, Sandra Rossi, Daniel Rueckert, Martin Rusnák, Juan Sahuquillo, Oliver Sakowitz, Renan Sanchez-Porras, Janos Sandor, Nadine Schäfer, Silke Schmidt, Herbert Schoechl, Guus Schoonman, Rico Frederik Schou, Elisabeth Schwendenwein, Charlie Sewalt, Ranjit D. Singh, Toril Skandsen, Peter Smielewski, Abayomi Sorinola, Emmanuel Stamatakis, Simon Stanworth, Robert Stevens, William Stewart, Nino Stocchetti, Nina Sundström, Riikka Takala, Viktória Tamás, Tomas Tamosuitis, Mark Steven Taylor, Braden Te Ao, Olli Tenovuo, Alice Theadom, Aurore Thibaut, Matt Thomas, Dick Tibboel, Marjolijn Timmers, Christos Tolias, Tony Trapani, Cristina Maria Tudora, Andreas Unterberg, Peter Vajkoczy, Egils Valeinis, Shirley Vallance, Zoltán Vámos, Mathieu van der Jagt, Joukje van der Naalt, Gregory Van der Steen, Jeroen T.J.M. van Dijck, Inge A. van Erp, Thomas A. van Essen, Wim Van Hecke, Caroline van Heugten, Dominique Van Praag, Ernest van Veen, Roel van Wijk, Thijs Vande Vyvere, Alessia Vargiolu, Emmanuel Vega, Kimberley Velt, Jan Verheyden, Paul M. Vespa, Anne Vik, Rimantas Vilcinis, Victor Volovici, Nicole von Steinbüchel, Daphne Voormolen, Peter Vulekovic, Daniel Whitehouse, Eveline Wiegers, Guy Williams, Stefan Wolf, Zhihui Yang, Peter Ylén, Alexander Younsi, Frederick A. Zeiler, Agate Ziverte, Tommaso Zoerle, Opeolu Adeoye, Neeraj Badjatia, Jason Barber, Michael Bergin, Kim Boase, Yelena Bodien, Randall Chesnut, John Corrigan, Karen Crawford, Ramon Diaz-Arrastia, Sureyya Dikmen, Ann-Christine Duhaime, Richard Ellenbogen, Venkata Feeser, Adam R. Ferguson, Brandon Foreman, Etienne Gaudette, Joseph Giacino, Luis Gonzalez, Shankar Gopinath, Ramesh Grandhi, Rao Gullapalli, Claude Hemphill, Gillian Hotz, Russell Huie, Ruchira Jha, Dirk C. Keene, Ryan Kitagawa, Frederick Korley, Joel Kramer, Natalie Kreitzer, Harvey Levin, Chris Lindsell, Joan Machamer, Christopher Madden, Alastair Martin, Thomas McAllister, Michael McCrea, Randall Merchant, Pratik Mukherjee, Lindsay Nelson, Laura B. Ngwenya, Florence Noel, Amber Nolan, David Okonkwo, Eva Palacios, Daniel Perl, Ava Puccio, Miri Rabinowitz, Claudia Robertson, Richard Ben Rodgers, Eric Rosenthal, Angelle Sander, Danielle Sandsmark, Andrea Schneider, David Schnyer, Seth Seabury, Mark Sherer, Gabriella Sugar, Nancy Temkin, Arthur Toga, Abel Torres-Espin, Alex Valadka, Mary Vassar, Kevin Wang, Vincent Wang, John K. Yue, Esther Yuh, Ross Zafonte

**Affiliations:** a Estonian Genome Center, Institute of Genomics, University of Tartu, Tartu, Estonia; b Division of Anaesthesia, University of Cambridge, Addenbrooke's Hospital, Cambridge, United Kingdom; c Division of Psychology, University of Stirling, Stirling, United Kingdom; d Division of Human Genetics, Department of Psychiatry, Yale University School of Medicine, New Haven, CT, USA; e Department of Psychiatry, Veterans Affairs Connecticut Healthcare Center, West Haven, CT, USA; f Center for Genomic Medicine, Massachusetts General Hospital, Boston, MA, USA; g McCance Center for Brain Health, Massachusetts General Hospital, Boston, MA, USA; h Broad Institute of MIT and Harvard, Cambridge, MA, USA; i Department of Biomedical Data Sciences, Leiden University Medical Center, Leiden, the Netherlands; j MRC Biostatistics Unit, Cambridge Institute of Public Health, University of Cambridge, Cambridge, United Kingdom; k Biostatistics Research Center, Herbert Wertheim School of Public Health and Human Longevity Science, University of California, San Diego, La Jolla, CA, USA; l Institute for Molecular Medicine Finland (FIMM), HiLIFE, University of Helsinki, Helsinki, Finland; m Department of Public Health, Faculty of Medicine, University of Helsinki, Helsinki, Finland; n Brain and Spinal Cord Injury Center, Zuckerberg San Francisco General Hospital and Trauma Center, San Francisco, CA, USA; o Department of Neurological Surgery, University of California, San Francisco, San Francisco, CA, USA; p Department of Neurosurgery, Antwerp University Hospital, Edegem, Belgium; q Department of Translational Neuroscience, Faculty of Medicine and Health Science, University of Antwerp, Antwerp, Belgium; r Department of Psychiatry, University of California, San Diego, La Jolla, CA, USA; s School of Public Health, University of California, San Diego, La Jolla, CA, USA; t VA San Diego Healthcare System, San Diego, CA, USA

**Keywords:** Traumatic brain injury, Mental health, Post-traumatic stress disorder, Depression, Polygenic risk score

## Abstract

**Background:**

Post-traumatic stress disorder (PTSD) and depression are common after mild traumatic brain injury (mTBI), but their biological drivers are uncertain. We therefore explored whether polygenic risk scores (PRS) derived for PTSD and major depressive disorder (MDD) are associated with the development of cognate TBI-related phenotypes.

**Methods:**

Meta-analyses were conducted using data from two multicenter, prospective observational cohort studies of patients with mTBI: the CENTER-TBI study (ClinicalTrials.gov ID NCT02210221) in Europe (December 2014–December 2017) and the TRACK-TBI study in the US (March 2014–July 2018). In both cohorts, the most common causes of injury were road traffic accidents and falls. Primary outcomes, specifically probable PTSD and depression, were defined at 6 months post-injury using scores ≥33 on the PTSD Checklist-5 and ≥15 on the Patient Health Questionnaire-9, respectively. We calculated PTSD-PRS and MDD-PRS for patients aged ≥17 years who had a Glasgow Coma Scale score of 13–15 upon hospital arrival and assessed their association with PTSD and depression following TBI. We also evaluated the transferability of the findings in a cohort of African Americans.

**Findings:**

Overall, 11.8% (219/1869) and 6.7% (124/1869) patients were classified as having probable PTSD and depression, respectively. The PTSD-PRS was significantly associated with higher adjusted odds of PTSD in both cohorts, with a pooled odds ratio (OR) of 1.55 [95% confidence interval (CI) 1.30–1.84, *p* < 0.001, *I*^*2*^ = 20.8%]. Although the MDD-PRS increased the risk of depression after TBI, it did not reach significance in the individual cohorts. However, in a combined analysis, the risk was significantly elevated with a pooled OR of 1.26 [95% CI 1.03–1.53, *p* = 0.02, *I*^*2*^ = 0%]. The addition of PRSs improved the proportion of outcome variance explained in the two study cohorts from 19.5% and 30.3% to 21.6% and 34.0% for PTSD; and from 11.0% and 22.5% to 12.8% and 22.6% for depression. Patients in the highest cognate PRS quintile had increased odds of 3.16 [95% CI 1.80–5.55] and 2.03 [95% CI 1.04–3.94] of developing PTSD or depression compared to the lowest quintile, respectively.

**Interpretation:**

Associations of PRSs with PTSD and depression following TBI are not disorder-specific. However, the overlap between MDD-PRS and depression following TBI is less robust compared to PTSD-PRS and PTSD. PRSs could improve risk prediction, and permit enrichment for interventional trials.

**Funding:**

This study was supported by funding by an FP7 grant from the 10.13039/501100000780European Union, 10.13039/501100007731Hannelore Kohl Stiftung, 10.13039/100009006Integra LifeSciences Corporation, NeuroTrauma Sciences, US 10.13039/100000002National Institutes of Health, US 10.13039/100000005Department of Defense, National Football League Advisory Board, US 10.13039/100000015Department of Energy, and 10.13039/100018727One Mind.

**Research in context**

**Evidence before this study**

We searched PubMed for articles (including original research and systematic reviews and meta-analyses published until August 1, 2023) exploring the genetic associations of post-traumatic stress disorder (PTSD) and depression following traumatic brain injury (TBI) using the following terms (“genetic” OR “genomics” OR “genome-wide association study” OR “GWAS” OR “polygenic risk score” OR “PRS”) AND (“traumatic brain injury” OR “TBI”) AND (“post-traumatic stress disorder” OR “PTSD” OR “depression”). Additional references were checked from the citation lists of papers identified using this search. We found no genome-wide association studies for PTSD and depression following TBI, and only one single-centre study using a polygenic risk score specifically in the context of TBI.

**Added value of this study**

Our demonstration that polygenic risk scores (PRS) for major depressive disorder (MDD) and PTSD are associated with the development of depression and PTSD after TBI, both with cognate and non-cognate TBI-related psychological health, speaks to the shared genetic vulnerability for adverse psychological health outcomes across specific diagnoses, replicated across two large cohort studies in TBI.

**Implications of all the available evidence**

Our results confirm that PTSD and depression following TBI have biological as well as environmental drivers, and that the genetic vulnerability to adverse psychological outcomes following TBI are shared across diagnoses. These findings could improve prediction of individual risk in prognostication, and permit enrichment of populations for trials of existing or new therapies.

## Introduction

Traumatic brain injury (TBI) poses a substantial burden to individuals, families, and societies.^1^ Patients with mild TBI (mTBI; defined as a Glasgow Coma Scale (GCS) score of 13–15) represent over 80% of cases, and a large proportion (∼50%) fail to recover completely by six months post-injury.^1^ In the prospective TRACK-TBI study, civilians with mTBI exhibited a significantly higher rate of probable major depressive disorder (MDD) than orthopaedic trauma controls at three months (8.8% vs. 3.0%) but not at six months.^2^ In the same study, the 6-month rate of probable post-traumatic stress disorder (PTSD) was 19.2% in the mTBI group and 9.8% in the orthopaedic control group. Similarly, another sample demonstrated high rates of persistent affective and other mTBI-related symptoms one year post-mTBI, despite a relatively complete recovery of cognitive performance and functional abilities.^3^

A survey of U.S. Army soldiers returning from deployment to Iraq found PTSD rates of 27.3% among those with mTBI, 16.2% among those with other injuries, and 9.1% among those with no injury.^4^ A prospective cohort study of civilians who sustained traumatic injuries found that individuals with mTBI were more likely to develop PTSD, with an odds ratio of 1.92.^5^

A prospective longitudinal cohort study utilised hospital-based patient registry data from a tertiary academic medical centre to compare individuals without head injuries to age-, sex-, and race-frequency-matched patients with mTBI. One year after the initial encounter, the hazard ratio for depression in the mTBI group was 3.9 (95% confidence interval (CI) 3.0–4.9) and for anxiety disorder (presumably including PTSD, though not separated out) was 2.9 (95% CI 2.4–3.6).^6^

Risk factors for these adverse psychological health outcomes are well recognised (including female sex, a history of mental health issues, prior TBI, and TBI caused by violence or assaults),^2^ but explain less than 10% of the variance in the risk of experiencing such outcomes following TBI. It would be useful to better understand the risk factors and mechanisms that predispose to these outcomes following TBI, since better understanding of their biological underpinnings in this context may allow more rational approaches to identifying, testing, and using therapeutic interventions.

One approach to elucidating the mechanisms underlying these processes would be to seek genetic associations that predict risk of their development. Such information could also provide information on the relative contributions of host vulnerability (as distinct from injury characteristics) in predisposing to these outcomes. Genome-wide association studies (GWAS) implicate multiple single nucleotide polymorphisms (SNPs) in PTSD^7^^,^^8^ and in MDD occurring in the absence of TBI.^9^^,^^10^ A pooled expression of risk posed by these SNPs can be expressed as polygenic risk scores (PRS) which better predict disease phenotypes than single SNPs.^11^ PRSs have been used to estimate the genetically determined risk of both MDD^12^ and PTSD.^13^^,^^14^ The interactions of environmental insults such as trauma with genetic susceptibility are well recognised in psychiatric disorders.^15^, ^16^, ^17^ More generally, it is recognised that environmental insults may be required to uncover biological susceptibility (such as seen with smoking and lung disease). However, well-established prediction models for a range of outcomes from TBI^18^^,^^19^ primarily focus on injury characteristics. While, for mTBI, the impact of pre-existing psychological health is recognised,^20^ none of these prediction models explicitly acknowledge or account for genetic susceptibility.

Given this context, it is interesting that we have recently shown that PRS can improve risk prediction of incident PTSD following mTBI.^21^ While these results are intriguing, several issues need to be addressed. First, given the relatively small sample size in our original study (*n* = 714),^21^ these results would benefit from robust replication. Second, it is important to determine if PRSs for MDD are similarly related to the risk of depression following TBI, and, if results were positive, undertake a replication of these findings. Finally, it is important to determine whether the relationship between PRSs for PTSD and depression are diagnosis-specific for their cognate psychological health phenotypes, or whether both represent an overall increase in the genetic risk of adverse psychological health outcomes, with associations that were blurred across the two diagnostic categories.

Overall, there remains substantial uncertainty about the relative contributions of injury severity and characteristics, impaired cognitive reserve, and genetic predisposition to the development of psychopathology after TBI. Disentangling these contributions is important to understand pathophysiology, identify therapeutic targets, and select enriched populations of patients who are more likely to respond to therapies aimed at such targets.

This study examines whether established PRSs for PTSD and MDD are associated with PTSD and depression following mTBI, explores whether associations with increased risk parcellate specifically with phenotype and cognate PRS, and undertakes cross replication and meta-analysis of findings from two large studies, one from Europe and one from the USA.

## Methods

### Study design

Participants were included from two prospective observational cohort studies: the Collaborative European NeuroTrauma Effectiveness Research in TBI (CENTER-TBI; ClinicalTrials.gov ID NCT02210221) study in Europe and Israel^22^; and the Transforming Research and Clinical Knowledge in TBI (TRACK-TBI) study in the USA.^23^ The CENTER-TBI study recruited 4509 patients between December 2014 and December 2017,^24^ while the TRACK-TBI study recruited 2697 patients between March 2014 and July 2018.^25^ Patients recruited to both studies presented with TBI within 24 h of injury, were triaged for an initial computed tomography (CT) scan, and had study consent available. Patients were excluded if they had a severe pre-existing neurological condition. The current analysis included patients genetically similar to the reference population from Europe (EUR) and Africa (AFR), aged ≥17 years, triaged to undergo CT, with a GCS score on hospital arrival of 13–15, with PTSD Checklist-5 (PCL-5) and Patient Health Questionnaire-9 (PHQ-9) scores at 6 months post-injury, and with genotyping array data permitting the calculation of PRSs. The TRACK-TBI patients included in this analysis were part of a previous study of PTSD following mTBI.^21^ However, the current analysis integrates them in a meta-analysis with CENTER-TBI patients and extends the analysis to include depression outcome, as well as replicating the associations in an African American cohort. The selection of subjects is shown in Supplementary Figure S1. From the CENTER-TBI cohort, 42 individuals genetically similar to non-European reference populations were excluded (4 African, 22 Latino, and 16 Asian). In the TRACK-TBI cohort, 497 individuals were identified as genetically similar to non-European reference populations. Among these, 188 African American participants were included in the analysis, while 309 patients were excluded (138 Latino, 32 Asian, and 139 from other origins such as Alaskan, Oceanian, Filipino, and mixed populations). This study followed the Strengthening the Reporting of Genetic Association Studies (STREGA) reporting guidelines.

### Measures

Clinical and demographic data, including the sex of study participants, were recorded by investigators at presentation (<24 h after TBI). Severity of brain injury was categorised using baseline GCS.^26^ Extracranial injuries were classified using the Abbreviated Injury Scale.^27^ A self-reported history of psychiatric illness was recorded for separate disorders and summarised as present or absent.

### Outcomes

Mental health outcomes were assessed using self-report questionnaires that are commonly used to screen for potential disorders. Definitive diagnosis of mental health disorders, e.g., within the framework of the Diagnostic and Statistical Manual-5 (DSM-5), requires a clinical interview, which was not part of the study.

The PCL-5 consists of 20 items covering four clusters of symptoms that characterise PTSD in DSM-5.^28^ The assessment has a total score of 0–80, and a score of 33 or more was used as a cut-off to identify probable PTSD.

The PHQ-9 consists of 9 items that are common symptoms of depression and yields a total score from 0 to 27. A cut-off of 15 or more was used to indicate probable MDD.^29^^,^^30^ These instruments and threshold scores have been widely used, with free translations in multiple languages,^31^ and are recommended by the NIH-NINDS Common data elements scheme for TBI outcomes,^32^^,^^33^ and in the case of PHQ-9, by the Common Measures in Mental Health Science Initiative (which did not consider PTSD).^34^

### Genotypes

Genotyping was performed using the Illumina Global Screening Array (GSA-24v2-0 + Multi-Disease). A standardised quality control process was applied across cohorts, excluding individuals with a call rate <97%, discrepancies between reported and genotype-based sex, and extreme heterozygosity (±3 standard deviation (SD) from the cohort mean). Autosomal variants were filtered by call rate (<97%), Hardy–Weinberg equilibrium (p < 1 × 10^−6^), minor allele frequency (<1%), and strand ambiguity (C/G or T/A polymorphisms) prior to imputation. Genotypes were imputed with the Haplotype Reference Consortium^35^ (release 1.1) imputation panel. Detailed quality control and imputation methods for CENTER-TBI and TRACK-TBI have been described previously.^36^

### Polygenic risk scores

Polygenic risk scores for PTSD (PTSD-PRS) and major depressive disorder (MDD-PRS) were calculated using PRS-CS (PRS-CS-auto, version 2021-01-04),^37^ which infers posterior SNP effect sizes under continuous shrinkage priors based on GWAS summary statistics and an external LD reference panel (503 European samples in 1000 Genomes Project) using HapMap3 variants.

To estimate SNP effect sizes for polygenic scores, we used GWAS summary statistics from the United States VA Million Veteran Program^38^^,^^39^ (MVP) of individuals genetically similar to the reference population from Europe. Over 1/3 of MVP participants have TBI^40^ and PTSD was defined as a total score of a 17-item self-report measure of past-month PTSD symptoms (PCL-Total GWAS, *n* = 186,689).^38^ Depression case–control status in the MVP cohort was determined based on an algorithm using an International Classification of Diseases codes captured in electronic health records, and meta-analysed with the UK Biobank, the Psychiatric Genomics Consortium, and FinnGen (*n* = 846,913).^39^ 23andMe data was excluded. PRSs were standardised separately in each cohort (CENTER-TBI, TRACK-TBI EUR, and TRACK-TBI AFR) to a mean of 0 and SD of 1.

### Statistics

CENTER-TBI and TRACK-TBI studies were compared using a *t*-test for mean age and Pearson's chi-square test for categorical variables. Spearman's correlations were calculated for mental health total scores and Pearson's correlations for the PRSs.

To analyse the association between PRS and mental health outcomes, mixed-effects logistic regression models with random intercepts for each study centre were employed. These analyses were conducted using the ‘glmer’ function from the R package ‘lme4’. Models were adjusted for age, sex, cause of injury, pre-injury psychiatric illness (yes or no), prior TBI (yes or no), and the first five ancestral principal components (PCs). Age was incorporated as a categorical variable (17–39, 40–64, or 65–90 years) to account for its potential non-linear effects related to the type of injury (Supplementary Table S1). We examined the performance of each PRS separately, and also the two in combination to determine associations with PTSD and depression following TBI. Adjusted odds ratios (aOR) and corresponding 95% confidence intervals are reported. aORs represent the odds for developing a given psychological health outcome per standardised unit increase in PRS, and pooled ORs (pORs) represent the adjusted odds for developing a given psychological health outcome across the two cohorts. Patients with missing data were excluded from multivariable analysis models. The performance of multivariable models was assessed using the conditional *R*^*2*^, calculated via the ‘r.squaredGLMM’ function from the ‘MuMIn’ package in R and area under the receiver operating characteristic curve (AUC). Sensitivity analyses were performed using linear mixed-effects models to assess the effect of PRS and baseline risk factors on PCL-5 and PHQ-9 total scores.

Fixed-effects model with inverse-variance weights was used for estimating pORs and 95% CIs using the ‘rma’ function in R package ‘metafor’.^41^ Pooled AUC values and 95% CIs across multiple studies were calculated assuming fixed effects and implemented in R package ‘metamisc’.^42^ Statistical heterogeneity was assessed by Cochran's *Q* test and *I*^*2*^ statistic.

In order to demonstrate that concordant results were obtained in each study, results are presented separately for each study and in addition, whenever possible, for pooled analyses across studies (the exceptions being where regulatory barriers prevented an appropriate level of data sharing). A two-sided *p*-value <0.05 was considered to indicate statistical significance. All statistical analyses were conducted using R (version 4.2.2).^43^

### Ethics

Ethical approval was obtained for each centre in accordance with local laws and procedures, and written informed consent was obtained from each participant, or from an ethically approved representative, enrolled in accordance with each approved protocol. Details of ethical committees granting approvals and approval numbers for CENTER-TBI institutions are provided on the CENTER-TBI website (https://www.center-tbi.eu/project/ethical-approval). Ethical approval for the TRACK-TBI study was provided by the San Francisco General Hospital Panel Institutional Review Board (IRB #12-09465; Reference #313687).

### Role of funding source

The funders had no role in the study design, data collection, data analysis, interpretation, or writing of the report^.^

## Results

Characteristics of patients with mTBI included in the analysis, compared with patients who were excluded, are shown in Supplementary Table S2. A total of 1143 (65.7% males) patients were included from CENTER-TBI and 726 (64.7% males) patients from TRACK-TBI (total *n* = 1869; Table 1). The mean age was 50.6 (SD 17.7) years in CENTER-TBI and 44.5 (SD 18.2) in TRACK-TBI. In both cohorts, road traffic accidents and falls were the most common causes of injury. The cohorts differed in the presence of pre-injury psychiatric illness (29.2% in TRACK-TBI vs. 11.3% CENTER-TBI; *p* < 0.001).

**Table 1:** Demographic and clinical characteristics of the two study cohorts.

	CENTER-TBI (*n* = 1143)	TRACK-TBI (*n* = 726)	*p-*value^a^
**Age (years)**			<0.001
Mean (SD)	50.6 (17.7)	44.5 (18.2)	
Median (IQR)	53 (36–64)	43 (28–59)	
17–39	331 (29.0%)	334 (46.0%)	
40–64	529 (46.3%)	269 (37.1%)	
65–90	283 (24.8%)	123 (16.9%)	
**Sex**			0.71
Female	392 (34.3%)	256 (35.3%)	
Male	751 (65.7%)	470 (64.7%)	
**Care pathway**			0.03
Emergency Room	335 (29.3%)	202 (27.8%)	
Admitted to hospital	534 (46.7%)	311 (42.8%)	
Intensive Care Unit	274 (24.0%)	213 (29.3%)	
**Cause of injury**			<0.001
Road traffic accident	469 (41.5%)	370 (51.1%)	
Fall	520 (46.0%)	246 (34.0%)	
Violence/assault	41 (3.6%)	21 (2.9%)	
Other	100 (8.8%)	87 (12.0%)	
Missing/unknown	13	2	
**GCS score at baseline**			0.003
13	82 (7.2%)	26 (3.6%)	
14	192 (16.8%)	141 (19.4%)	
15	869 (76.0%)	559 (77.0%)	
**Pre-injury psychiatric illness**			<0.001
Absent	985 (86.7%)	514 (70.8%)	
Present	151 (13.3%)	212 (29.2%)	
Missing	7	0	
**Prior TBI**			<0.001
Absent	956 (87.1%)	524 (76.7%)	
Present	142 (12.9%)	159 (23.3%)	
Missing	45	43	

SD, standard deviation; IQR, interquartile range; GCS, Glasgow Coma Scale; TBI, traumatic brain injury.

a CENTER-TBI and TRACK-TBI studies are compared using a *t*-test for mean age and Pearson's chi-square test for categorical variables.

Among analysed patients with mTBI, there were more PTSD and MDD patients 6-months after the injury in TRACK-TBI (PCL-5 ≥ 33: 16.2% in TRACK-TBI vs. 9.2% in CENTER-TBI, *p* < 0.001; PHQ-9 ≥ 15: 8.4% in TRACK-TBI vs. 5.6% in CENTER-TBI, *p* = 0.02; Table 2).

**Table 2:** Summary statistics of mental health outcomes for study cohorts.

Outcome	CENTER-TBI (*n* = 1143)	TRACK-TBI (*n* = 726)	*p-*value^a^
**PCL-5 Total score**			
≥33 (n, %)	103 (9.2%)	116 (16.2%)	<0.001
Median (IQR)	7 (2–17)	9 (3–22)	
Missing	19	12	
**PHQ-9 Total score**			
≥15 (n, %)	63 (5.6%)	61 (8.4%)	0.02
Median (IQR)	3 (1–7)	3 (1–7)	
Missing	16	4	

IQR, interquartile range; PCL-5, Post-traumatic Stress Disorder Checklist-5; PHQ-9, Patient Health Questionnaire-9.

a CENTER-TBI and TRACK-TBI studies are compared using Pearson's chi-square test.

Consistent with previous studies, there were strong relationships between the PHQ-9 and PCL-5 total scores for the mental health assessments (*r* = 0.71, *p* < 0.001 in CENTER-TBI; *r* = 0.69, *p* < 0.001 in TRACK-TBI), and moderate relationships between PTSD and MDD PRSs (*r* = 0.37, *p* < 0.001 in CENTER-TBI; *r* = 0.43, *p* < 0.001 in TRACK-TBI).

**Fig. 1:**
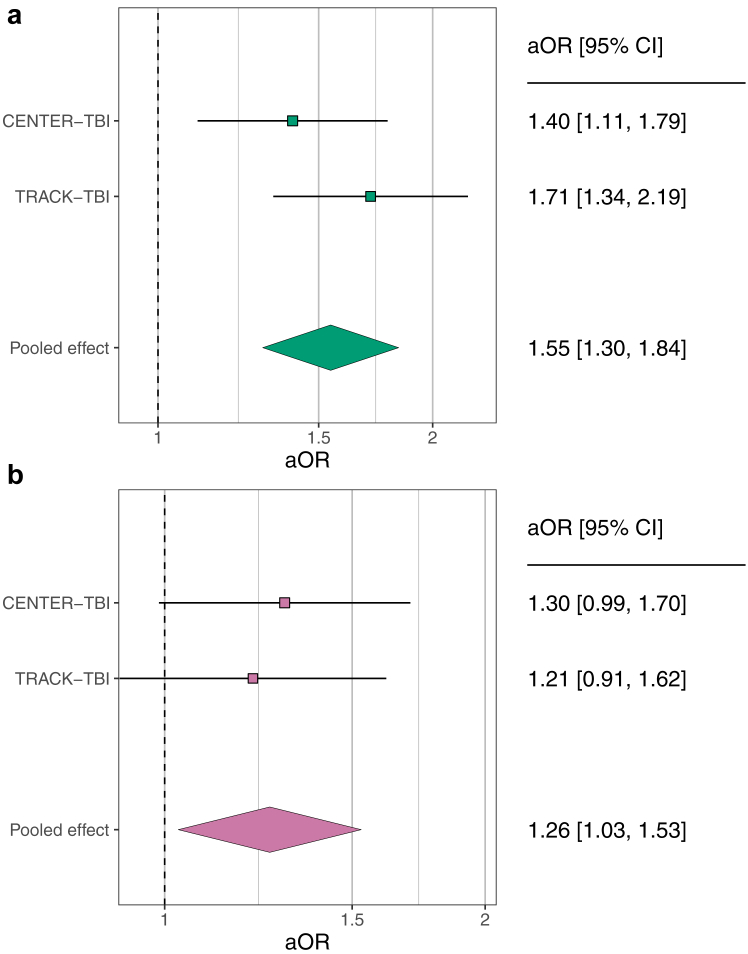
**Forest plots of PRS associations with PTSD and depression following TBI 6 months post-TBI.** The multivariable-adjusted odds ratios (aORs) and 95% CIs together with the pooled effect using a fixed-effects meta-analysis are presented for **(a)** PTSD-PRS on PTSD and **(b)** MDD-PRS on depression following TBI.

PTSD-PRS in a multivariable model showed a significant association with increased adjusted odds of PTSD at 6 months post-injury in both cohorts with a pOR of 1.55 (95% CI 1.30–1.84, *p* < 0.001, *I*^*2*^ = 20.8%; Fig. 1a; Table 3, model 1). The baseline model of PTSD, adjusted for demographic and clinical features, and ancestry PCs, showed a pooled AUC of 0.774 (95% CI 0.740–0.804). The proportion of variance explained by the fixed and random effects was 19.5% (CENTER-TBI) and 30.3% (TRACK-TBI). The addition of PRS improved pooled AUC further to 0.790 (95% CI 0.757–0.820), and the variance explained to 21.6% (CENTER-TBI) and 34.0% (TRACK-TBI) (Supplementary Figures S2a and c; Supplementary Table S3).

**Table 3:** Multivariable logistic regression analyses for mental health outcomes after 6 months of injury.

Outcome	Model	PRS used in the model	CENTER-TBI		TRACK-TBI		Pooled analysis	
			OR (95% CI)	*p*-value	OR (95% CI)	*p*-value	OR (95% CI)	*p*-value
PCL-5 ≥ 33	1	PTSD-PRS	1.40 (1.11–1.79)	0.006	1.71 (1.34–2.19)	<0.001	1.55 (1.30–1.84)	<0.001
	2	MDD-PRS	1.21 (0.97–1.52)	0.09	1.30 (1.03–1.65)	0.03	1.25 (1.07–1.47)	0.006
	3	PTSD-PRS	1.36 (1.05–1.75)	0.02	1.67 (1.28–2.19)	<0.001	1.50 (1.24–1.80)	<0.001
		MDD-PRS	1.10 (0.86–1.39)	0.45	1.06 (0.82–1.38)	0.66	1.08 (0.91–1.29)	0.39
PHQ-9 ≥ 15	4	MDD-PRS	1.30 (0.99–1.70)	0.06	1.21 (0.91–1.62)	0.20	1.26 (1.03–1.53)	0.02
	5	PTSD-PRS	1.49 (1.11–1.99)	0.008	1.56 (1.15–2.11)	0.004	1.52 (1.23–1.88)	<0.001
	6	MDD-PRS	1.15 (0.85–1.54)	0.36	1.01 (0.73–1.40)	0.93	1.08 (0.87–1.35)	0.48
		PTSD-PRS	1.41 (1.03–1.93)	0.03	1.55 (1.11–2.16)	0.01	1.47 (1.17–1.85)	0.001

OR, odds ratio; CI, confidence interval; PRS, polygenic risk score; PTSD, post-traumatic stress disorder; MDD, major depressive disorder; PCL-5, Post-traumatic Stress Disorder Checklist-5; PHQ-9, Patient Health Questionnaire-9.

Results are provided separately for each study and for the pooled meta-analysis. Each model estimates the impact of PRS on one mental health outcome, with all models adjusted for age, sex, cause of injury, prior TBI, psychiatric history, and five principal components. Models 1–3 assess the associations with PTSD and Models 4–6 with depression following TBI.

**Fig. 2:**
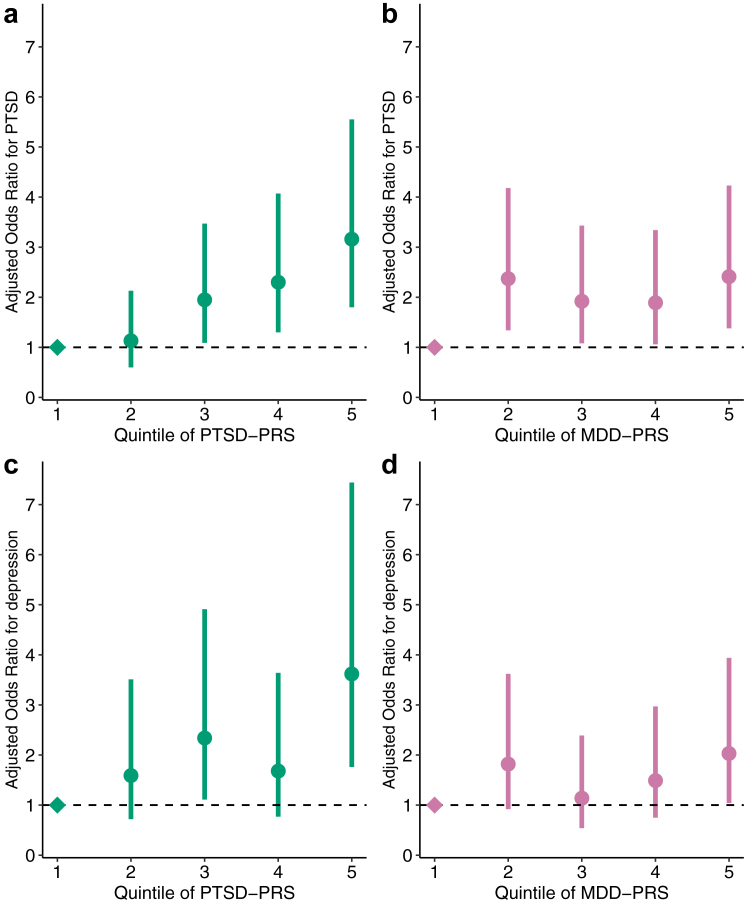
**Adjusted odds ratios (aOR) for each quintile of the PRS for PTSD and depression following TBI.** aOR and 95% CI are calculated relative to TBI patients with PRS in the lowest quintile. Quantiles are plotted for cognate PRSs **(a)** PTSD phenotype vs. PTSD-PRS and **(d)** depression following TBI phenotype vs. MDD-PRS, and for non-cognate PRSs **(b)** PTSD phenotype vs. MDD-PRS and **(c)** depression following TBI phenotype vs. PTSD-PRS.

There was also a statistically significant relationship between MDD-PRS and PTSD (pOR = 1.25, 95% CI 1.07–1.47, *p* = 0.006, *I*^*2*^ = 0%; Table 3, model 2). When both PRSs were assessed jointly, the PTSD-PRS remained significantly associated with a PTSD outcome, while the MDD-PRS did not (Table 3, model 3; Supplementary Table S4). The incidence of PTSD scaled with the PTSD-PRS, such that, when compared to patients in the lowest quintile, patients in the top quintile had a pOR of 3.16 (95% CI 1.80–5.55) of developing PTSD (Fig. 2a; Supplementary Table S5).

While MDD-PRS increased the risk of developing depression following TBI, this association did not achieve significance in either individual cohort (Supplementary Table S3). However, a combined analysis across both cohorts showed that the MDD-PRS resulted in a significantly increased risk of depression following TBI, with a pOR of 1.26 (95% CI 1.03–1.53, *p* = 0.02, *I*^*2*^ = 0%; Fig. 1b; Table 3, model 4). Adjusted for demographic and clinical characteristics, and PCs, the baseline model of TBI-related depression yielded a pooled AUC value of 0.741 (95% CI 0.694–0.783) with the proportion of variance explained of 11.0% (CENTER-TBI) and 22.5% (TRACK-TBI). Subsequently, the addition of PRS further increased the pooled AUC to 0.749 (95% CI 0.702–0.791) and the proportion of outcome variance explained improved to 12.8% in the CENTER-TBI and to 22.6% in the TRACK-TBI cohort, respectively (Supplementary Figures S2b and d; Supplementary Table S4).

The PTSD-PRS was also associated with the risk of depression following TBI with a pOR of 1.52 (95% CI 1.23–1.88, *p* < 0.001, *I*^*2*^ = 0%; Table 3, model 5). When both PRSs were assessed jointly, the association of post-TBI depression with the PTSD-PRS remained significant (pOR = 1.47, 95% CI 1.17–1.85, *p* = 0.001, *I*^*2*^ = 0%), but the association with the MDD-PRS failed to retain significance (pOR = 1.08, 95% CI 0.87–1.35, *p* = 0.48, *I*^*2*^ = 0%; Table 3, model 6).

Unlike the association seen between PTSD and PTSD-PRS, the incidence of post-TBI depression did not scale with increase of MDD-PRS (Fig. 2d). Further, a comparison between the lowest and the highest quintiles of the PTSD-PRS and MDD-PRS in modelling post-TBI depression showed a higher pOR for the PTSD-PRS (3.62, 95% CI 1.76–7.44 vs. 2.03, 95% CI 1.04–3.94), although the confidence intervals for the two overlapped substantially (Supplementary Table S5). We found no evidence of heterogeneity between studies (Supplementary Table S6).

Sensitivity analyses using PCL-5 and PHQ-9 total scores showed that both PTSD-PRS and PTSD, as well as MDD-PRS and depression following TBI, were statistically significantly associated (Supplementary Tables S7 and S8). In a cohort of African Americans (Supplementary Table S9), the association between PTSD-PRS and depression following TBI was replicated. However, PTSD was found to be more strongly linked to MDD-PRS rather than PTSD-PRS (Supplementary Tables S10 and S11). Supplementary Figure S3 illustrates the distribution of PRSs across the two reference populations.

## Discussion

Established risk factors for adverse psychological health outcomes following TBI include female sex, TBI caused by violence or assaults, prior TBI, and a history of mental health issues,^2^ reflected in the fact that a higher incidence of past psychiatric illness in the TRACK-TBI cohort (29.2% vs. 13.3%) was associated with higher rate of both PTSD (16.2% vs. 9.2%) and depression (8.4% vs. 5.6%) when compared to the CENTER-TBI cohort. However, these risk factors account for only a fraction of the variance in the risk of experiencing such outcomes. Our results, replicated across cohorts, suggest that even when known risk factors are accounted for, there may be genetic predisposition to adverse psychological health outcomes after TBI.

We were able to demonstrate these associations with the increased sample size afforded by combining the CENTER-TBI and TRACK-TBI datasets, which prospectively collected data using the NIH/NINDS Common Data Elements scheme,^44^ best illustrated by the association of MDD-PRS with post-TBI depression which only reached significance in the combined analysis. The magnitude of the associations with PRSs was comparable to that reported in studies conducted in non-TBI settings, even after adjusting for prior psychological health.^13^^,^^45^

The incidence of PTSD increased with higher PTSD-PRS, but TBI-related depression did not scale with increase of MDD-PRS. These discordant findings were in keeping with an examination of the association between PTSD and TBI-related depression with non-cognate PRSs (i.e., PTSD with MDD-PRS and TBI-related depression with PTSD-PRS)—both of which were significant. These cross-diagnosis relationships are consistent with the latent structure of mental disorders, which consistently show that PTSD and MDD load together on a Distress Disorders subfactor of Internalizing Disorders,^46^ implying a high degree of shared vulnerability to these two conditions.^47^^,^^48^

However, models which included both PRSs showed that the association between MDD-PRS and PTSD outcome no longer achieved significance. Surprisingly, in a similar combined model, the association between PTSD-PRS and TBI-related depression remained significant, while that between MDD-PRS and TBI-related depression did not. These results suggest that the PTSD-PRS provides a better estimate of biological vulnerability to post-TBI depression than the MDD-PRS.

Several potential explanations can be offered for this unexpected finding. First, many patients may have suffered from both PTSD and TBI-related depression, and the shared phenotype may have been better captured by the assessment tool used for PTSD (PCL-5) than that used for depression (PHQ-9). Indeed, of the 13.7% (*n* = 256) of our overall cohort of 1869 patients who were likely experiencing PTSD or depression using these instruments, 34.0% (*n* = 87) had both conditions. Second, while the thresholds that we selected on each instrument to define cases were based on past literature,^28^, ^29^, ^30^ the classification of TBI-related depression may have been either too lenient or stringent to optimally seek associations with genetic risk. Exploration of patients with isolated diagnoses of PTSD and depression, and/or the exploration of alternative thresholds might address these issues, but our sample size was not large enough to do this.

However, even with the PHQ-9 threshold we used for classification, TBI-related depression does not appear to be less genetically driven than MDD, since we were able to show significant associations between this phenotype and the PTSD-PRS. Indeed, in past studies, MDD associated with traumatic events shows increased heritability compared to non-trauma-related MDD,^47^ and genetic loading for PTSD was significantly associated with reporting trauma in individuals with MDD.^48^ It is therefore possible that MDD in the absence of trauma may be biologically different from depression seen in the context of TBI (and possibly other trauma), and hence poorly predicted by a PRS derived from non-TBI-related MDD, and less responsive to conventional pharmacotherapy used in MDD.^49^^,^^50^ This hypothesis is supported by a recent publication showing that abnormalities in network connectivity in functional magnetic resonance imaging studies of TBI-related depression are different from those seen in MDD,^51^ and are similar to those that predict poor response to conventional antidepressant pharmacotherapy,^52^^,^^53^ and responsiveness to neuromodulation using non-pharmacological interventions.^54^ Our data strengthen the view that conventional MDD and TBI-related depression are not identical, but may have different genetic, phenotypic, and therapeutic attributes.

We demonstrated that, even when using GWAS data from individuals genetically similar to European reference populations, the distributions of PTSD-PRS and MDD-PRS between Europeans and non-Europeans showed substantial overlap. Consequently, we were able to replicate the association between PTSD-PRS and depression following TBI, though not with PTSD, in an African American cohort. The greater variability in PTSD-PRS distributions between groups may account for this difference. With the growing availability of large multi-ancestry and non-European GWAS datasets, such as those from the MVP and the All of Us Research Program, we can now include SNPs more common in underrepresented ancestries. This facilitates the development of multi-ancestry PRSs for predicting risk of mental health outcomes following TBI.

Most health outcomes are viewed as the result of interactions between environmental factors and an individual's susceptibility to these factors.^55^ However, TBI has commonly been excluded from such conceptual constructs. In the TBI literature, the dominant view has traditionally been that the severity and location of the mechanical injury primarily determines outcomes—i.e., the functional, cognitive, and psychological sequelae. It has long been known that simple host factors like age can modify recovery, but the realisation that other aspects of host biology can also modulate injury outcomes is relatively new.

It is not yet well understood by what mechanisms genetic factors (as referenced by PRSs) for neuropsychiatric disorders increase risk for these disorders in the context of mTBI. The lack of detail available regarding prior psychological health in our datasets means that it is impossible to completely exclude unmasking of a pre-existing mental health condition. Further, even if the associations we demonstrate are not confounded by prior psychological health, linkages between PRS and highly polygenic phenotypes such as PTSD and depression may have complex and variable substrates. These include direct or indirect genetic susceptibility, interactions between genes and environmental insults (TBI in our case), or associations between genes and environment that have no genetic causal basis.^56^

Additional questions relate to mechanisms for our results. It has been suggested that mTBI may diminish the capacity to employ cognitive resources that would normally be engaged in problem-solving and regulating emotions after trauma, thereby increasing susceptibility to PTSD and related problems such as MDD.^57^ It would follow, then, that those individuals at increased genetic risk for these disorders would be most likely to develop mental disorders in the context of mTBI. It is also worth noting that a recent GWAS study of TBI occurrence determined that TBI occurrence has a heritable basis and is genetically correlated to risk-taking behaviours and several psychiatric disorders, notably PTSD.^40^ These observations raise the possibility of shared genetic risk for exposure to TBI and these frequently observed psychiatric sequelae. Future work that attempts to disaggregate these various genetic risk factors will be important in advancing our understanding of these associations between mTBI and mental disorders such as PTSD and MDD.

Critically, a large proportion of this enormous burden of disability following mTBI arises from adverse psychological health outcomes, including PTSD and depression. Effective early interventions are available for both conditions in other contexts,^58^^,^^59^ and could be strong candidates for evaluation (and eventually, established therapy) in TBI. While these PRSs are not yet of clinical utility, the primary contribution of our paper is the demonstration that there is a genetic component to the risk for these mental health outcomes following TBI, and that this is shared with these disorders when they occur outside of the context of TBI. Future larger multi-ancestry GWAS, or exome or whole genome sequencing, might generate PRSs consisting of common and rare variants that could explain even more variance than the current versions.

Our multicenter, longitudinal, prospective design, the large number of participants in comparison to most previous genetic association studies in TBI, replication and meta-analysis across two large studies, and the use of multivariable statistical analysis that incorporated non-genetic predictors in addition to PRS, are all strengths of the study.

We acknowledge clear limitations. All patients were aged over 17 years, mostly recruited from large trauma centres, and required a head CT based on local protocols—all of which constrain severity and case-mix. Patients lost to six-month follow-up were excluded from the analysis, and such missingness seemed to be non-random as revealed by significant differences in key characteristics (e.g., cause of injury and prior TBI in CENTER-TBI and age, psychiatric history, CT scans, major extracranial injury, and prior TBI in TRACK-TBI; Supplementary Table S2) between included and excluded patient groups. Our self-reported label of pre-existing psychiatric history is subject to recall and reporting bias and lacks detailed information regarding the specific type of psychiatric illness. The instruments we used to identify PTSD and depression (PCL-5 and PHQ-9) have been widely used, but still do not fully replicate the gold standard diagnosis resulting from an interview by an experienced clinician. In the current dataset, no specific diagnosis is provided for pre-injury psychological conditions. While comparisons with non-TBI participants from previous studies^2^, ^3^, ^4^, ^5^ suggest that the mental health outcomes under investigation are likely linked to TBI, we cannot definitively confirm this causal relationship. Currently, calculated PRSs are primarily applicable to individuals genetically similar to the reference population from Europe. A future priority is to extend these findings to other ancestral groups to promote greater diversity in genetic research and prevent the exacerbation of health disparities among different populations.^60^ We have not included non-psychiatric comorbidities in these analyses and need to recognise that significant systemic illness may represent an unmeasured confounder in our analyses. Finally, since we only included patients with a GCS score of 13–15, these results only apply to mild TBI presenting to hospital. Additional studies will be needed to replicate these results in moderate and severe TBI,^61^ or patients with TBI who do not present to hospital, such as individuals with sport-related concussion.

Despite these limitations, the findings we provide are important. They suggest that PTSD and depression following TBI may have biological as well as environmental drivers, and that the genetic risks we demonstrate are associated with development (or worsening) of these mental health phenotypes after TBI. The association that we demonstrate with existing PRSs (and subsequent improvements in these scores)^11^ may allow some refinement of individual risk in prognostication and could permit enrichment of populations for trials of existing or new therapies. Finally, these findings may allow exploration of the early use of antidepressants following TBI. One option, if genetic data are available, might be a secondary analysis of data from current trials of antidepressant medication in TBI.^62^

## Contributors

The study was conceptualised by MK, LW, MBS, and DKM; based on data collected in the CENTER-TBI and TRACK-TBI studies (see group authorship statements); funding and resources were obtained by GTM, AIRM, and DKM; data curation was undertaken by MK, LW, LP, FH, and XS; formal analysis was undertaken by MK and LW, with additional input from DFL, LP, FH, and XS, and supervision by EWS, SRich, SJ, AP, SRip, JR, MRB, and DKM. The initial draft was produced by MK, LW, MBS and DKM, and critically reviewed and revised by all authors. MK, LW, FH, and XS accessed and verified the underlying data in the study. All authors read and approved the final version of the manuscript and take responsibility for the decision to submit it for publication.

## Data sharing statement

All the outputs from the current analysis have been included in this manuscript and supplementary materials, so no unpublished results exist. Access to individual patient data is available by application to the respective studies—both for CENTER-TBI (https://www.center-tbi.eu/data) and TRACK-TBI (https://tracktbi.ucsf.edu/collaboration-opportunities).

## Declaration of interests

LW reports receiving consultancy fees from NeuroTrauma Sciences and Spaulding-Harvard TBI Model System outside the submitted work. JR declares payment for expert testimony from the National Football League. GTM has received funding from NeuroTrauma Sciences and One Mind. AIRM serves as an advisory board member for PressuraNeuro and declares consulting fees from Novartis and NeuroTrauma Sciences. MBS serves as a data and safety monitoring board member for the University of Nebraska and the University of Boston, declares royalties with UpToDate, stock options in Oxeia Biopharmaceuticals, and consulting fees from Acadia Pharmaceuticals, Aptinyx, atai Life Sciences, BigHealth, Biogen, Bionomics, BioXcel Therapeutics, Boehringer Ingelheim, Clexio, Delix Therapeutics, Eisai, EmpowerPharm, Engrail Therapeutics, Janssen, Jazz Pharmaceuticals, NeuroTrauma Sciences, PureTech Health, Sage Therapeutics, Sumitomo Pharma, and Roche/Genentech. DKM declares research collaborations or consultancy/lecture fees with regard to the following organisations: NeuroTrauma Sciences, Lantmannen AB, GlaxoSmithKline Ltd., PresSura Neuro, CSL Behring, Invex Ltd., and Integra Neurosciences Ltd. The remaining authors declare no competing interests.

## Appendix A Supplementary data

Supplementary material

Group members

## References

[1] Maas A.I.R., Menon D.K., Manley G.T. (2022). Traumatic brain injury: progress and challenges in prevention, clinical care, and research. Lancet Neurol.

[2] Stein M.B., Jain S., Giacino J.T. (2019). Risk of posttraumatic stress disorder and major depression in civilian patients after mild traumatic brain injury. JAMA Psychiatry.

[3] Dikmen S., Machamer J., Temkin N. (2017). Mild traumatic brain injury: longitudinal study of cognition, functional status, and post-traumatic symptoms. J Neurotrauma.

[4] Hoge C.W., McGurk D., Thomas J.L., Cox A.L., Engel C.C., Castro C.A. (2008). Mild traumatic brain injury in U.S. Soldiers returning from Iraq. N Engl J Med.

[5] Bryant R.A., O'Donnell M.L., Creamer M., McFarlane A.C., Clark C.R., Silove D. (2010). The psychiatric sequelae of traumatic injury. Am J Psychiatry.

[6] Izzy S., Chen P.M., Tahir Z. (2022). Association of traumatic brain injury with the risk of developing chronic cardiovascular, endocrine, neurological, and psychiatric disorders. JAMA Netw Open.

[7] Nievergelt C.M., Maihofer A.X., Klengel T. (2019). International meta-analysis of PTSD genome-wide association studies identifies sex- and ancestry-specific genetic risk loci. Nat Commun.

[8] Stein M.B., Chen C.Y., Ursano R.J. (2016). Genome-wide association studies of posttraumatic stress disorder in two cohorts of US army soldiers. JAMA Psychiatry.

[9] Kendall K.M., Assche E.V., Andlauer T.F.M. (2021). The genetic basis of major depression. Psychol Med.

[10] Als T.D., Kurki M.I., Grove J. (2023). Depression pathophysiology, risk prediction of recurrence and comorbid psychiatric disorders using genome-wide analyses. Nat Med.

[11] Wray N.R., Lin T., Austin J. (2021). From basic science to clinical application of polygenic risk scores: a primer. JAMA Psychiatry.

[12] Kosciuszko M., Steptoe A., Ajnakina O. (2023). Genetic propensity, socioeconomic status, and trajectories of depression over a course of 14 years in older adults. Transl Psychiatry.

[13] Waszczuk M.A., Docherty A.R., Shabalin A.A. (2022). Polygenic prediction of PTSD trajectories in 9/11 responders. Psychol Med.

[14] Campbell-Sills L., Papini S., Norman S.B. (2023). Associations of polygenic risk scores with posttraumatic stress symptom trajectories following combat deployment. Psychol Med.

[15] Assary E., Vincent J.P., Keers R., Pluess M. (2018). Gene-environment interaction and psychiatric disorders: review and future directions. Semin Cell Dev Biol.

[16] Park Y.M., Shekhtman T., Kelsoe J.R. (2020). Interaction between adverse childhood experiences and polygenic risk in patients with bipolar disorder. Transl Psychiatry.

[17] Chuong M., Adams M.J., Kwong A.S.F., Haley C.S., Amador C., McIntosh A.M. (2022). Genome-by-Trauma exposure interactions in adults with depression in the UK Biobank. JAMA Psychiatry.

[18] MCT CRASH Trial Collaborators (2008). Predicting outcome after traumatic brain injury: practical prognostic models based on large cohort of international patients. BMJ.

[19] Steyerberg E.W., Mushkudiani N., Perel P. (2008). Predicting outcome after traumatic brain injury: development and international validation of prognostic scores based on admission characteristics. PLoS Med.

[20] Mikolić A., Steyerberg E.W., Polinder S. (2023). Prognostic models for global functional outcome and post-concussion symptoms following mild traumatic brain injury: a collaborative European NeuroTrauma effectiveness research in traumatic brain injury (CENTER-TBI) study. J Neurotrauma.

[21] Stein M.B., Jain S., Parodi L. (2023). Polygenic risk for mental disorders as predictors of posttraumatic stress disorder after mild traumatic brain injury. Transl Psychiatry.

[22] Collaborative European NeuroTrauma effectiveness research in TBI (CENTER-TBI). https://www.center-tbi.eu/.

[23] Transforming research and clinical knowledge in TBI (TRACK-TBI). https://tracktbi.ucsf.edu/transforming-research-and-clinical-knowledge-tbi.

[24] Steyerberg E.W., Wiegers E., Sewalt C. (2019). Case-mix, care pathways, and outcomes in patients with traumatic brain injury in CENTER-TBI: a European prospective, multicentre, longitudinal, cohort study. Lancet Neurol.

[25] Nelson L.D., Temkin N.R., Dikmen S. (2019). Recovery after mild traumatic brain injury in patients presenting to US level I trauma centers. JAMA Neurol.

[26] Ercole A., Dixit A., Nelson D.W. (2021). Imputation strategies for missing baseline neurological assessment covariates after traumatic brain injury: a CENTER-TBI study. PLoS One.

[27] Committee on Medical Aspects of Automotive Safety (1971). Rating the severity of tissue damage. JAMA.

[28] Blevins C.A., Weathers F.W., Davis M.T., Witte T.K., Domino J.L. (2015). The posttraumatic stress disorder checklist for DSM-5 (PCL-5): development and initial psychometric evaluation. J Trauma Stress.

[29] Kroenke K., Spitzer R.L., Williams J.B.W. (2001). The PHQ-9. J Gen Intern Med.

[30] Manea L., Gilbody S., McMillan D. (2012). Optimal cut-off score for diagnosing depression with the Patient Health Questionnaire (PHQ-9): a meta-analysis. CMAJ.

[31] CENTER-TBI//validated translations outcome instruments. https://www.center-tbi.eu/project/validated-translations-outcome-instruments.

[32] Nelson L.D., Ranson J., Ferguson A.R. (2017). Validating multi-dimensional outcome assessment using the traumatic brain injury common data elements: an analysis of the TRACK-TBI pilot study sample. J Neurotrauma.

[33] Wilson L., Horton L., Polinder S. (2022). Tailoring multi-dimensional outcomes to level of functional recovery after traumatic brain injury. J Neurotrauma.

[34] Farber G.K., Gage S., Kemmer D., White R. (2023). Common measures in mental health: a joint initiative by funders and journals. Lancet Psychiatry.

[35] McCarthy S., Das S., Kretzschmar W. (2016). A reference panel of 64,976 haplotypes for genotype imputation. Nat Genet.

[36] Kals M., Kunzmann K., Parodi L. (2022). A genome-wide association study of outcome from traumatic brain injury. eBioMedicine.

[37] Ge T., Chen C.Y., Ni Y., Feng Y.C.A., Smoller J.W. (2019). Polygenic prediction via Bayesian regression and continuous shrinkage priors. Nat Commun.

[38] Stein M.B., Levey D.F., Cheng Z. (2021). Genome-wide association analyses of post-traumatic stress disorder and its symptom subdomains in the million veteran program. Nat Genet.

[39] Levey D.F., Stein M.B., Wendt F.R. (2021). Bi-ancestral depression GWAS in the million veteran program and meta-analysis in >1.2 million individuals highlight new therapeutic directions. Nat Neurosci.

[40] Merritt V.C., Maihofer A.X., Gasperi M. (2024). Genome-wide association study of traumatic brain injury in U.S. military veterans enrolled in the VA million veteran program. Mol Psychiatry.

[41] Viechtbauer W. (2010). Conducting meta-analyses in R with the metafor package. J Stat Softw.

[42] Debray T.P., Damen J.A., Riley R.D. (2019). A framework for meta-analysis of prediction model studies with binary and time-to-event outcomes. Stat Methods Med Res.

[43] R Core Team R: a language and environment for statistical computing. https://www.R-project.org/.

[44] Traumatic brain injury | NINDS common data elements. https://www.commondataelements.ninds.nih.gov/Traumatic%20Brain%20Injury.

[45] Lipsky R.K., Garrett M.E., Dennis M.F. (2023). Impact of traumatic life events and polygenic risk scores for major depression and posttraumatic stress disorder on Iraq/Afghanistan Veterans. J Psychiatr Res.

[46] de Jonge P., Wardenaar K.J., Lim C.C.W. (2018). The cross-national structure of mental disorders: results from the World Mental Health Surveys. Psychol Med.

[47] Coleman J.R.I., Peyrot W.J., Purves K.L. (2020). Genome-wide gene-environment analyses of major depressive disorder and reported lifetime traumatic experiences in UK Biobank. Mol Psychiatry.

[48] Mundy J., Hübel C., Gelernter J. (2022). Psychological trauma and the genetic overlap between posttraumatic stress disorder and major depressive disorder. Psychol Med.

[49] Fann J.R., Bombardier C.H., Temkin N. (2017). Sertraline for major depression during the year following traumatic brain injury: a randomized controlled trial. J Head Trauma Rehabil.

[50] Fann J.R., Hart T., Schomer K.G. (2009). Treatment for depression after traumatic brain injury: a systematic review. J Neurotrauma.

[51] Siddiqi S.H., Kandala S., Hacker C.D. (2023). Precision functional MRI mapping reveals distinct connectivity patterns for depression associated with traumatic brain injury. Sci Transl Med.

[52] Chin Fatt C.R., Jha M.K., Cooper C.M. (2020). Effect of intrinsic patterns of functional brain connectivity in moderating antidepressant treatment response in major depression. Am J Psychiatry.

[53] Korgaonkar M.S., Goldstein-Piekarski A.N., Fornito A., Williams L.M. (2020). Intrinsic connectomes are a predictive biomarker of remission in major depressive disorder. Mol Psychiatry.

[54] Siddiqi S.H., Schaper F.L.W.V.J., Horn A. (2021). Brain stimulation and brain lesions converge on common causal circuits in neuropsychiatric disease. Nat Hum Behav.

[55] Virolainen S.J., VonHandorf A., Viel K.C.M.F., Weirauch M.T., Kottyan L.C. (2023). Gene–environment interactions and their impact on human health. Genes Immun.

[56] Raffington L., Mallard T., Harden K.P. (2020). Polygenic scores in developmental psychology: invite genetics in, leave Biodeterminism behind. Annu Rev Dev Psychol.

[57] Stein M.B., McAllister T.W. (2009). Exploring the convergence of posttraumatic stress disorder and mild traumatic brain injury. Am J Psychiatry.

[58] Bisson J.I., Wright L.A., Jones K.A. (2021). Preventing the onset of post traumatic stress disorder. Clin Psychol Rev.

[59] Coventry P.A., Meader N., Melton H. (2020). Psychological and pharmacological interventions for posttraumatic stress disorder and comorbid mental health problems following complex traumatic events: systematic review and component network meta-analysis. PLoS Med.

[60] Martin A.R., Kanai M., Kamatani Y., Okada Y., Neale B.M., Daly M.J. (2019). Clinical use of current polygenic risk scores may exacerbate health disparities. Nat Genet.

[61] Wilson L., Horton L., Kunzmann K. (2021). Understanding the relationship between cognitive performance and function in daily life after traumatic brain injury. J Neurol Neurosurg Psychiatry.

[62] Trial of sertraline to prevent post-traumatic brain injury depression. https://www.isrctn.com/ISRCTN17518945.

