# Supplementary material for: Genetic vulnerability and adverse mental health outcomes following mild traumatic brain injury: a meta-analysis of CENTER-TBI and TRACK-TBI cohorts
Source: eClinicalMedicine. 2024 Dec 5;78:102956. doi: 10.1016/j.eclinm.2024.102956 (PMC11667043; doi:10.1016/j.eclinm.2024.102956)

Supplementary material

Supplementary Table S1. Mental health outcomes by age groups 2

Supplementary Table S2. Demographic and clinical characteristics of mild TBI patients 3

Supplementary Table S3. Results of the mixed-effects logistic regression models for the mental health outcomes after 6 months of injury 4

Supplementary Table S4. Conditional R^2 and area under the ROC curve values of mixed-effects logistic regression models for mental health outcomes after 6 months of injury 6

Supplementary Table S5. Pooled odds ratio estimates and 95% CI of PTSD and depression following TBI by cognate and non-cognate PRS quintiles after 6 months of injury 7

Supplementary Table S6. Heterogeneity measures in meta-analysis 8

Supplementary Table S7. Sensitivity analysis on PCL-5 total score 9

Supplementary Table S8. Sensitivity analysis on PHQ-9 total score 10

Supplementary Table S9. Demographic and clinical characteristics of the TRACK-TBI patients by reference populations 11

Supplementary Table S10. Replication of post-traumatic stress disorder at 6 months post-injury in a cohort of African Americans (*n* = 188) 12

Supplementary Table S11. Replication of depression following TBI at 6 months post-injury in a cohort of African Americans (*n* = 188) 14

Supplementary Figure S1. Subject selection flow diagram 16

Supplementary Figure S2. Receiver operating characteristic (ROC) curves 17

Supplementary Figure S3. Distributions of standardized polygenic risk scores (PRSs) in the TRACK-TBI cohort by reference populations 18

Supplementary Table S1. Mental health outcomes by age groups.

	**CENTER-TBI (EUR, *n* = 1143)**		**TRACK-TBI (EUR, *n* = 726)**		**TRACK-TBI (AFR, *n* = 188)**	
**PCL-5 Total Score**	**≥33 (n, %)**	**Total (n)**	**≥33 (n, %)**	**Total (n)**	**≥33 (n, %)**	**Total (n)**
Age 17-39	30 (9.2%)	326	56 (17.2%)	326	56 (17.2%)	99
Age 40-64	58 (11.2%)	519	53 (19.9%)	267	53 (19.9%)	74
Age 65-90	15 (5.4%)	279	7 (5.8%)	121	7 (5.8%)	13
Age 17-90	103 (9.2%)	1124	116 (16.2%)	714	61 (8.4%)	186
**PHQ-9 Total Score**	**≥15 (n, %)**	**Total (n)**	**≥15 (n, %)**	**Total (n)**	**≥15 (n, %)**	**Total (n)**
Age 17-39	13 (4.0%)	327	28 (19.8%)	332	20 (19.8%)	101
Age 40-64	32 (6.1%)	523	9 (12.2%)	267	9 (12.2%)	74
Age 65-90	18 (6.5%)	277	2 (15.4%)	123	2 (15.4%)	13
Age 17-90	63 (5.6%)	1127	61 (8.4%)	722	31 (16.5%)	188

PCL-5 = Post-traumatic Stress Disorder Checklist-5; PHQ-9 = Patient Health Questionnaire-9.

Supplementary Table S2. Demographic and clinical characteristics of mild TBI patients. Comparison of patients with a baseline GCS of 13–15, included in the analysis, versus those excluded, such as patients with missing data (6-month mental health outcomes or genotype information) and genetically similar to non-European reference populations. The included and excluded groups were compared using a *t*-test for mean age and Pearson's chi-square test for categorical variables.

	CENTER-TBI			TRACK-TBI		
	**Included samples (n = 1143)**	**Excluded samples (n = 1601)**	***p*-value**	**Included samples (n = 726)**	**Excluded samples (n = 1249)**	***p*-value**
**Age (years)**			0.45			<0.001
Mean (SD)	50.6 (17.7)	51.2 (21.5)		44.5 (18.2)	39.6 (16.8)	
**Sex**			0.18			0.27
Female	392 (34.3%)	590 (36.9%)		256 (35.3%)	409 (32.7%)	
Male	751 (65.7%)	1011 (63.1%)		470 (64.7%)	840 (67.3%)	
**Care pathway**			0.77			0.20
Emergency Room	335 (29.3%)	473 (29.5%)		202 (27.8%)	306 (24.5%)	
Admitted to hospital	534 (46.7%)	763 (47.7%)		311 (42.8%)	541 (43.3%)	
Intensive Care Unit	274 (24.0%)	365 (22.8%)		213 (29.3%)	402 (32.2%)	
**Cause of injury**			<0.001			<0.001
Road traffic accident	469 (41.5%)	484 (30.8%)		370 (51.1%)	759 (61.0%)	
Fall	520 (46.0%)	799 (50.9%)		246 (34.0%)	288 (23.1%)	
Violence/assault	41 (3.6%)	140 (8.9%)		21 (2.9%)	105 (8.4%)	
Other	100 (8.8%)	148 (9.4%)		87 (12.0%)	93 (7.5%)	
Missing/unknown	13	30		2	4	
**GCS score at baseline**			0.17			0.26
13	82 (7.2%)	87 (5.4%)		26 (3.6%)	64 (5.1%)	
14	192 (16.8%)	272 (17.0%)		141 (19.4%)	230 (18.4%)	
15	869 (76.0%)	1242 (77.6%)		559 (77.0%)	955 (76.5%)	
**CT imaging abnormality**			0.06			0.03
Absent	577 (53.3%)	838 (57.1%)		430 (60.3%)	784 (65.3%)	
Present	506 (46.7%)	630 (42.9%)		283 (39.7%)	417 (34.7%)	
Missing/uninterpretable	60	133		13	48	
**Major extracranial injury[1]**			0.45			0.02
Absent	837 (73.2%)	1194 (74.6%)		625 (86.1%)	1025 (82.1%)	
Present	306 (26.8%)	407 (25.4%)		101 (13.9%)	224 (17.9%)	
**Pre-injury psychiatric illness**			0.63			<0.001
Absent	985 (86.7%)	1357 (86.0%)		514 (70.8%)	1024 (82.1%)	
Present	151 (13.3%)	221 (14.0%)		212 (29.2%)	224 (17.9%)	
Missing	7	23		0	1	
**Prior TBI**			0.01			0.22
Absent	956 (87.1%)	1381 (90.3%)		524 (76.7%)	893 (79.3%)	
Present	142 (12.9%)	148 (9.7%)		159 (23.3%)	233 (20.7%)	
Missing	45	72		43	123	

[1]Any non-head & neck Abbreviated Injury Score ≥ 3 (serious injury).
SD = standard deviation; IQR = interquartile range; GCS = Glasgow Coma Scale; CT = computed tomography; TBI = traumatic brain injury.

Supplementary Table S3. Results of the mixed-effects logistic regression models for the mental health outcomes after 6 months of injury.

Model 1: association between baseline features (age, sex, psychiatric history, prior TBI, cause of injury) and PTSD.

**Model 1**		**CENTER-TBI**		**TRACK-TBI**	
**Outcome**	**Variable**	**OR (95% CI)**	***p*-value**	**OR (95% CI)**	***p*-value**
PCL-5 ≥ 33	Age: 40–64	1.23 (0.75–2.04)	0.42	1.13 (0.71–1.81)	0.60
	Age: 65–90	0.58 (0.30–1.15)	0.12	0.32 (0.13–0.74)	0.008
	Sex: female	0.90 (0.57–1.43)	0.66	1.12 (0.69–1.80)	0.65
	Psychiatric history	3.69 (2.21–6.15)	<0.001	2.98 (1.88–4.75)	<0.001
	Prior TBI	1.28 (0.69–2.35)	0.43	1.83 (1.12–3.00)	0.02
	Injury cause: violence	2.53 (1.01–6.32)	0.05	3.34 (1.14–9.78)	0.03

CENTER-TBI: AUC = 0.764; conditional R^2 = 18.9%.
TRACK-TBI: AUC = 0.789; conditional R^2 = 28.9%.
Pooled effect: AUC = 0.777 (95% CI 0.745–0.806).

Model 2: association between baseline features and PTSD, adjusted for principal components.

**Model 2**		**CENTER-TBI**		**TRACK-TBI**	
**Outcome**	**Variable**	**OR (95% CI)**	***p*-value**	**OR (95% CI)**	***p*-value**
PCL-5 ≥ 33	Age: 40–64	1.29 (0.78–2.16)	0.32	1.10 (0.69–1.76)	0.69
	Age: 65–90	0.62 (0.31–1.24)	0.18	0.31 (0.13–0.73)	0.007
	Sex: female	0.98 (0.61–1.56)	0.92	1.10 (0.68–1.78)	0.69
	Psychiatric history	3.62 (2.17–6.04)	<0.001	3.07 (1.92–4.90)	<0.001
	Prior TBI	1.41 (0.76–2.62)	0.27	1.79 (1.09–2.95)	0.02
	Injury cause: violence	2.68 (1.05–6.82)	0.04	3.58 (1.20–10.65)	0.02
	PC1	1.72 (1.29–2.30)	<0.001	1.31 (0.83–2.09)	0.25
	PC2	0.75 (0.58–0.98)	0.03	0.94 (0.70–1.27)	0.71
	PC3	0.91 (0.68–1.22)	0.52	0.96 (0.84–1.10)	0.55
	PC4	0.98 (0.74–1.29)	0.88	0.96 (0.85–1.10)	0.58
	PC5	0.78 (0.63–0.97)	0.02	0.97 (0.88–1.07)	0.56

CENTER-TBI: AUC = 0.748; conditional R^2 = 19.5%.
TRACK-TBI: AUC = 0.794; conditional R^2 = 30.3%.
Pooled effect: AUC = 0.774 (95% CI 0.740–0.804).

Model 3: association between PTSD-PRS and PTSD, adjusted for baseline features and principal components.

**Model 3***		**CENTER-TBI**		**TRACK-TBI**	
**Outcome**	**Variable**	**OR (95% CI)**	***p*-value**	**OR (95% CI)**	***p*-value**
PCL-5 ≥ 33	PTSD-PRS	1.40 (1.11–1.79)	0.006	1.71 (1.34–2.19)	<0.001
	Age: 40–64	1.27 (0.76–2.12)	0.37	1.12 (0.69–1.81)	0.65
	Age: 65–90	0.62 (0.31–1.25)	0.18	0.34 (0.14–0.81)	0.02
	Sex: female	0.93 (0.58–1.49)	0.75	1.19 (0.73–1.95)	0.48
	Psychiatric history	3.44 (2.04–5.79)	<0.001	2.89 (1.79–4.67)	<0.001
	Prior TBI	1.48 (0.79–2.76)	0.22	1.71 (1.03–2.86)	0.04
	Injury cause: violence	2.41 (0.93–6.23)	0.07	4.72 (1.51–14.69)	0.008

*Model is adjusted for the first five ancestral principal components.
CENTER-TBI: AUC = 0.762; conditional R^2 = 21.6%. Likelihood ratio test (LRT) p = 0.005 compared to Model 2.
TRACK-TBI: AUC = 0.815; conditional R^2 = 34.0%. LRT p < 0.001 compared to Model 2.
Pooled effect: AUC = 0.790 (95% CI 0.757–0.820).

Model 4: association between baseline features and depression following TBI.

Model 4		CENTER-TBI		TRACK-TBI	
**Outcome**	**Variable**	**OR (95% CI)**	***p*-value**	**OR (95% CI)**	***p*-value**
PHQ-9 ≥ 15	Age: 40–64	1.47 (0.73–2.96)	0.28	1.20 (0.66–2.18)	0.56
	Age: 65–90	1.68 (0.78–3.66)	0.19	0.65 (0.25–1.69)	0.38
	Sex: female	1.54 (0.90–2.65)	0.12	1.40 (0.78–2.53)	0.26
	Psychiatric history	2.25 (1.18–4.29)	0.01	4.05 (2.25–7.28)	<0.001
	Prior TBI	1.86 (0.93–3.73)	0.08	1.64 (0.89–3.03)	0.11
	Injury cause: violence	1.15 (0.26–5.12)	0.86	2.80 (0.79–9.88)	0.11

CENTER-TBI: AUC = 0.721; conditional R^2 = 12.2%.
TRACK-TBI: AUC = 0.793; conditional R^2 = 21.9%.
Pooled effect: AUC = 0.758 (95% CI 0.712–0.798).

Model 5: association between baseline features and depression following TBI, adjusted for principal components.

Model 5		CENTER-TBI		TRACK-TBI	
**Outcome**	**Variable**	**OR (95% CI)**	***p*-value**	**OR (95% CI)**	***p*-value**
PHQ-9 ≥ 15	Age: 40–64	1.50 (0.74–3.03)	0.26	1.18 (0.64–2.16)	0.60
	Age: 65–90	1.67 (0.76–3.66)	0.20	0.67 (0.26–1.74)	0.41
	Sex: female	1.64 (0.95–2.82)	0.08	1.40 (0.77–2.55)	0.27
	Psychiatric history	2.01 (1.04–3.89)	0.04	4.00 (2.21–7.23)	<0.001
	Prior TBI	1.98 (0.99–3.99)	0.06	1.67 (0.90–3.10)	0.11
	Injury cause: violence	1.23 (0.28–5.51)	0.79	2.81 (0.78–10.12)	0.11
	PC1	1.51 (1.06–2.13)	0.02	0.87 (0.51-1.48)	0.61
	PC2	0.89 (0.65–1.22)	0.46	1.06 (0.72-1.54)	0.78
	PC3	0.95 (0.68–1.33)	0.77	1.00 (0.84-1.18)	0.99
	PC4	0.93 (0.68–1.28)	0.68	0.92 (0.78-1.09)	0.34
	PC5	1.03 (0.78–1.37)	0.83	0.93 (0.82-1.05)	0.25

CENTER-TBI: AUC = 0.686; conditional R^2 = 11.0%.
TRACK-TBI: AUC = 0.793; conditional R^2 = 22.5%.
Pooled effect: AUC = 0.741 (95% CI 0.694–0.783).

Model 6: association between MDD-PRS and depression following TBI, adjusted for baseline features and principal components.

Model 6*		CENTER-TBI		TRACK-TBI	
**Outcome**	**Variable**	**OR (95% CI)**	***p*-value**	**OR (95% CI)**	***p*-value**
PHQ-9 ≥ 15	MDD-PRS	1.30 (0.99–1.70)	0.06	1.21 (0.91–1.62)	0.20
	Age: 40–64	1.53 (0.75–3.10)	0.24	1.20 (0.65–2.20)	0.56
	Age: 65–90	1.71 (0.78–3.76)	0.18	0.68 (0.26–1.78)	0.44
	Sex: female	1.67 (0.97–2.88)	0.07	1.42 (0.78–2.57)	0.25
	Psychiatric history	1.95 (1.01–3.77)	0.05	3.89 (2.15–7.04)	<0.001
	Prior TBI	2.03 (1.01–4.09)	0.05	1.63 (0.88–3.04)	0.12
	Injury cause: violence	1.15 (0.26–5.16)	0.85	2.91 (0.81–10.51)	0.10

*Model is adjusted for the first five ancestral principal components.
CENTER-TBI: AUC = 0.702; conditional R^2 = 12.8%. LRT p = 0.06 compared to Model 5.
TRACK-TBI: AUC = 0.794; conditional R^2 = 22.6%. LRT p = 0.20 compared to Model 5.
Pooled effect: AUC = 0.749 (95% CI 0.702–0.791).

OR = odds ratio; CI = confidence interval; PRS = polygenic risk score; PTSD = post-traumatic stress disorder; MDD = major depressive disorder; PCL-5 = Post-traumatic Stress Disorder Checklist-5; PHQ-9 = Patient Health Questionnaire-9; AUC = area under the receiver operating characteristic curve; PC = principal component; TBI = traumatic brain injury.

Supplementary Table S4. Conditional R^2 and area under the ROC curve values of mixed-effects logistic regression models for mental health outcomes after 6 months of injury.

		**Conditional R^2**		**AUC**		
**Outcome**	**PRS used in the model***	**CENTER-TBI**	**TRACK-TBI**	**CENTER-TBI**	**TRACK-TBI**	**Pooled analysis (95% CI)**
PCL-5 ≥ 33	PTSD-PRS	0.216	0.340	0.762	0.815	0.790 (0.757–0.820)
	MDD-PRS	0.203	0.310	0.762	0.802	0.783 (0.751–0.813)
	PTSD-PRS MDD-PRS	0.218	0.340	0.767	0.816	0.792 (0.760–0.821)
PHQ-9 ≥ 15	MDD-PRS	0.128	0.226	0.702	0.794	0.749 (0.702–0.791)
	PTSD-PRS	0.149	0.252	0.717	0.802	0.756 (0.710–0.797)
	MDD-PRS PTSD-PRS	0.152	0.252	0.718	0.803	0.757 (0.711–0.798)

*Models are adjusted for age, sex, psychiatric history, prior TBI, cause of injury, and the first five ancestral principal components.
AUC = area under the receiver operating characteristic curve; PRS = polygenic risk score; CI = confidence interval; PTSD = post-traumatic stress disorder; MDD = major depressive disorder; PCL-5 = Post-traumatic Stress Disorder Checklist-5; PHQ-9 = Patient Health Questionnaire-9.

Supplementary Table S5. Pooled odds ratio estimates and 95% CI of PTSD and depression following TBI by cognate and non-cognate PRS quintiles after 6 months of injury. Pooled odds ratios are calculated relative to the lowest quintile.

PRS quintile	PTSD-PRS for PTSD	MDD-PRS for PTSD	MDD-PRS for depression following TBI	PTSD-PRS for depression following TBI
**1**	1 (ref)	1 (ref)	1 (ref)	1 (ref)
**2**	1.13 (0.60–2.13)	2.37 (1.34–4.18)	1.82 (0.92–3.62)	1.59 (0.72–3.51)
**3**	1.95 (1.09–3.47)	1.92 (1.08–3.43)	1.14 (0.54–2.39)	2.34 (1.11–4.91)
**4**	2.30 (1.30–4.07)	1.89 (1.06–3.34)	1.49 (0.75–2.97)	1.68 (0.77–3.64)
**5**	3.16 (1.80–5.55)	2.41 (1.38–4.23)	2.03 (1.04–3.94)	3.62 (1.76–7.44)

CI = confidence interval; PRS = polygenic risk score; PTSD = post-traumatic stress disorder; MDD = major depressive disorder.

Supplementary Table S6. Heterogeneity measures in meta-analysis. I^2 statistic and heterogeneity *p*-value computed from Cochran's *Q* statistic.

		**Heterogeneity measure**	
**Outcome**	**PRS used in the model***	**I^2 statistic**	***p*-value**
PCL-5 ≥ 33	PTSD-PRS	20.8%	0.26
	MDD-PRS	0%	0.68
	PTSD-PRS<br>MDD-PRS	16.6%<br>0%	0.27<br>0.85
PHQ-9 ≥ 15	MDD-PRS	0%	0.73
	PTSD-PRS	0%	0.82
	MDD-PRS<br>PTSD-PRS	0%<br>0%	0.57<br>0.68

*Models are adjusted for age, sex, psychiatric history, prior TBI, cause of injury, and the first five ancestral principal components.

PRS = polygenic risk score; PTSD = post-traumatic stress disorder; MDD = major depressive disorder; PCL-5 = Post-traumatic Stress Disorder Checklist-5; PHQ-9 = Patient Health Questionnaire-9.

Supplementary Table S7. Sensitivity analysis on PCL-5 total score.

<table>
<tr><th></th><th></th><th colspan="3">CENTER-TBI</th><th colspan="3">TRACK-TBI</th></tr>
<tr><th>Outcome</th><th>Variable</th><th>Estimate</th><th>SE</th><th>p-value</th><th>Estimate</th><th>SE</th><th>p-value</th></tr>
<tr><td rowspan="7">PCL-5 total score</td><td>PTSD-PRS</td><td>1.11</td><td>0.40</td><td>0.006</td><td>2.77</td><td>0.61</td><td><0.001</td></tr>
<tr><td>Age: 40–64</td><td>0.77</td><td>0.90</td><td rowspan="2">0.03</td><td>0.61</td><td>1.31</td><td rowspan="2">0.007</td></tr>
<tr><td>Age: 65–90</td><td>-1.79</td><td>1.05</td><td>-4.60</td><td>1.68</td></tr>
<tr><td>Sex: female</td><td>1.44</td><td>0.80</td><td>0.07</td><td>3.14</td><td>1.28</td><td>0.01</td></tr>
<tr><td>Psychiatric history</td><td>7.16</td><td>1.38</td><td><0.001</td><td>7.45</td><td>1.33</td><td><0.001</td></tr>
<tr><td>Prior TBI</td><td>2.06</td><td>1.16</td><td>0.07</td><td>2.87</td><td>1.42</td><td>0.04</td></tr>
<tr><td>Injury cause: violence</td><td>4.91</td><td>2.05</td><td>0.02</td><td>11.70</td><td>3.54</td><td>0.001</td></tr>
</table>

Model is adjusted for the first five ancestral principal components.

SE = standard error of the estimate; TBI = traumatic brain injury.

CENTER-TBI: conditional R^2 = 12.1%. Likelihood ratio test (LRT) $p = 0.005$ compared to model without PTSD-PRS.

TRACK-TBI: conditional R^2 = 19.9%. LRT $p < 0.001$ compared to model without PTSD-PRS.

Supplementary Table S8. Sensitivity analysis on PHQ-9 total score.

		CENTER-TBI			TRACK-TBI		
**Outcome**	**Variable**	**Estimate**	**SE**	***p*-value**	**Estimate**	**SE**	***p*-value**
PHQ-9 total score	MDD-PRS	0.43	0.15	0.003	0.44	0.20	0.03
	Age: 40–64	0.19	0.35	0.54	0.40	0.44	0.07
	Age: 65–90	-0.22	0.41		-0.92	0.56	
	Sex: female	1.09	0.31	<0.001	1.17	0.42	0.006
	Psychiatric history	3.31	0.44	<0.001	3.54	0.44	<0.001
	Prior TBI	0.66	0.45	0.14	0.99	0.47	0.04
	Injury cause: violence	0.39	0.80	0.62	2.98	1.18	0.01

Model is adjusted for the first five ancestral principal components.
SE = standard error of the estimate; TBI = traumatic brain injury.
CENTER-TBI: conditional R^2 = 9.9%. Likelihood ratio test (LRT) p = 0.003 compared to model without MDD-PRS.
TRACK-TBI: conditional R^2 = 18.4%. LRT p = 0.03 compared to model without MDD-PRS.

Supplementary Table S9. Demographic and clinical characteristics of the TRACK-TBI patients by reference populations. European (EUR, *n* = 726) and African American (AFR, *n* = 188) individuals were compared using a *t*-test for mean age and Pearson's chi-square test for categorical variables.

	TRACK-TBI (EUR, *n* = 726)	TRACK-TBI (AFR, *n* = 188)	*p*-value
**Age (years)**			0.004
Mean (SD)	44.5 (18.2)	39.8 (15.0)	
17-39	334 (46.0%)	101 (53.7%)	
40-64	269 (37.1%)	74 (39.4%)	
65-90	123 (16.9%)	13 (6.9%)	
**Sex**			0.93
Female	256 (35.3%)	67 (35.6%)	
Male	470 (64.7%)	121 (64.4%)	
**Care pathway**			0.02
Emergency Room	202 (27.8%)	59 (31.4%)	
Admitted to hospital	311 (42.8%)	93 (49.5%)	
Intensive Care Unit	213 (29.3%)	36 (19.1%)	
**Cause of injury**			<0.001
Road traffic accident	370 (51.1%)	117 (62.9%)	
Fall	246 (34.0%)	33 (17.7%)	
Violence/assault	21 (2.9%)	23 (12.4%)	
Other	87 (12.0%)	13 (7.0%)	
Missing/unknown	2	2	
**GCS score at baseline**			0.04
13	26 (3.6%)	10 (5.3%)	
14	141 (19.4%)	23 (12.2%)	
15	559 (77.0%)	155 (82.5%)	
**Pre-injury psychiatric illness**			<0.001
Absent	514 (70.8%)	157 (83.5%)	
Present	212 (29.2%)	31 (16.5%)	
**Prior TBI**			0.77
Absent	524 (76.7%)	135 (75.4%)	
Present	159 (23.3%)	44 (24.6%)	
Missing	43	9	
**PCL-5 Total Score**			<0.001
≥33 (n, %)	116 (16.2%)	65 (34.9%)	
Missing	12	2	
**PHQ-9 Total Score**			0.002
≥15 (n, %)	61 (8.4%)	31 (16.5%)	
Missing	4	0	

SD = standard deviation; IQR = interquartile range; GCS = Glasgow Coma Scale; CT = computed tomography; TBI = traumatic brain injury.

Supplementary Table S10. Replication of post-traumatic stress disorder at 6 months post-injury in a cohort of African Americans (*n* = 188).

Model 1: association between baseline features and PTSD, adjusted for principal components.

Model 1		TRACK-TBI (AFR)	
**Outcome**	**Variable**	**OR (95% CI)**	***p*-value**
PCL-5 ≥ 33	Age: 40–64	0.72 (0.34–1.53)	0.39
	Age: 65–90	0.20 (0.03–1.26)	0.09
	Sex: female	1.12 (0.51–2.49)	0.78
	Psychiatric history	4.29 (1.52–12.07)	0.006
	Prior TBI	4.12 (1.65–10.27)	0.002
	Injury cause: violence	2.19 (0.73–6.53)	0.16
	PC1	0.87 (0.60–1.25)	0.44
	PC2	1.18 (0.74–1.91)	0.49
	PC3	0.86 (0.69–1.07)	0.19
	PC4	0.94 (0.77–1.15)	0.56
	PC5	1.01 (0.85–1.21)	0.88

AUC = 0.799 (95% CI 0.732–0.866); conditional R^2 = 31.6%.

Model 2: association between PTSD-PRS and PTSD, adjusted for baseline features and principal components.

Model 2*		TRACK-TBI (AFR)	
**Outcome**	**Variable**	**OR (95% CI)**	***p*-value**
PCL-5 ≥ 33	PTSD-PRS	2.93 (0.68–12.73)	0.15
	Age: 40–64	0.72 (0.34–1.56)	0.41
	Age: 65–90	0.20 (0.03–1.28)	0.09
	Sex: female	1.08 (0.48–2.40)	0.85
	Psychiatric history	3.71 (1.30–10.59)	0.01
	Prior TBI	4.54 (1.78–11.57)	0.002
	Injury cause: violence	2.15 (0.72–6.40)	0.17

*Model is adjusted for the first five ancestral principal components.
AUC = 0.795 (95% CI 0.726–0.864); conditional R^2 = 32.9%.

Model 3: association between MDD-PRS and PTSD, adjusted for baseline features and principal components.

Model 3*		TRACK-TBI (AFR)	
**Outcome**	**Variable**	**OR (95% CI)**	***p*-value**
PCL-5 ≥ 33	MDD-PRS	1.67 (1.10–2.53)	0.02
	Age: 40–64	0.82 (0.37–1.80)	0.62
	Age: 65–90	0.16 (0.02–1.15)	0.07
	Sex: female	1.15 (0.51–2.59)	0.73
	Psychiatric history	4.03 (1.40–11.63)	0.01
	Prior TBI	4.61 (1.79–11.86)	0.002
	Injury cause: violence	1.93 (0.63–5.90)	0.25

*Model is adjusted for the first five ancestral principal components.
AUC = 0.828 (95% CI 0.766–0.891); conditional R^2 = 36.4%.

Model 4: association between PTSD-PRS, MDD-PRS, and PTSD, adjusted for baseline features and principal components.

**Model 4***		**TRACK-TBI (AFR)**	
**Outcome**	**Variable**	**OR (95% CI)**	***p*-value**
PCL-5 ≥ 33	PTSD-PRS	1.70 (0.35–8.33)	0.52
	MDD-PRS	1.59 (1.02–2.47)	0.04
	Age: 40–64	0.81 (0.37–1.77)	0.59
	Age: 65–90	0.16 (0.02–1.20)	0.07
	Sex: female	1.13 (0.50–2.55)	0.77
	Psychiatric history	3.80 (1.30–11.09)	0.01
	Prior TBI	4.78 (1.84–12.39)	0.001
	Injury cause: violence	1.95 (0.64–5.93)	0.24

*Model is adjusted for the first five ancestral principal components.
AUC = 0.824 (95% CI 0.761–0.824); conditional R^2 = 36.5%.

OR = odds ratio; CI = confidence interval; PRS = polygenic risk score; PTSD = post-traumatic stress disorder; MDD = major depressive disorder; PCL-5 = Post-traumatic Stress Disorder Checklist-5; PHQ-9 = Patient Health Questionnaire-9; AUC = area under the receiver operating characteristic curve; PC = principal component; TBI = traumatic brain injury.

Supplementary Table S11. Replication of depression following TBI at 6 months post-injury in a cohort of African Americans (*n* = 188).

Model 1: association between baseline features and depression following TBI, adjusted for principal components.

Model 1		TRACK-TBI (AFR)	
**Outcome**	**Variable**	**OR (95% CI)**	***p*-value**
PCL-5 ≥ 33	Age: 40–64	0.66 (0.24–1.81)	0.42
	Age: 65–90	1.21 (0.20–7.19)	0.84
	Sex: female	1.09 (0.42–2.84)	0.86
	Psychiatric history	6.96 (2.53–19.13)	<0.001
	Prior TBI	2.08 (0.76–5.68)	0.15
	Injury cause: violence	0.47 (0.09–2.52)	0.38
	PC1	0.91 (0.58–1.44)	0.69
	PC2	1.21 (0.67–2.21)	0.53
	PC3	1.15 (0.88–1.52)	0.31
	PC4	1.00 (0.78–1.28)	1
	PC5	1.11 (0.90–1.38)	0.33

AUC = 0.757 (95% CI 0.637–0.876); conditional R^2 = 24.4%.

Model 2: association between MDD-PRS and depression following TBI, adjusted for baseline features and principal components.

Model 2*		TRACK-TBI (AFR)	
**Outcome**	**Variable**	**OR (95% CI)**	***p*-value**
PCL-5 ≥ 33	MDD-PRS	1.14 (0.70–1.84)	0.60
	Age: 40–64	0.69 (0.25–1.90)	0.47
	Age: 65–90	1.19 (0.20–7.18)	0.85
	Sex: female	1.10 (0.42–2.87)	0.84
	Psychiatric history	6.78 (2.46–18.73)	<0.001
	Prior TBI	2.13 (0.77–5.90)	0.14
	Injury cause: violence	0.46 (0.09–2.46)	0.37

*Model is adjusted for the first five ancestral principal components.
AUC = 0.755 (95% CI 0.640–0.870); conditional R^2 = 24.6%.

Model 3: association between PTSD-PRS and depression following TBI, adjusted for baseline features and principal components.

Model 3*		TRACK-TBI (AFR)	
**Outcome**	**Variable**	**OR (95% CI)**	***p*-value**
PCL-5 ≥ 33	PTSD-PRS	6.99 (1.11–44.10)	0.04
	Age: 40–64	0.62 (0.22–1.75)	0.37
	Age: 65–90	1.27 (0.21–7.73)	0.80
	Sex: female	1.09 (0.41–2.86)	0.87
	Psychiatric history	5.81 (2.08–16.27)	<0.001
	Prior TBI	2.20 (0.78–6.23)	0.14
	Injury cause: violence	0.52 (0.10–2.78)	0.44

*Model is adjusted for the first five ancestral principal components.
AUC = 0.778 (95% CI 0.668–0.887); conditional R^2 = 29.7%.

Model 4: association between MDD-PRS, PTSD-PRS, and depression following TBI, adjusted for baseline features and principal components.

**Model 4***		**TRACK-TBI (AFR)**	
**Outcome**	**Variable**	**OR (95% CI)**	***p*-value**
PCL-5 ≥ 33	MDD-PRS	0.96 (0.57–1.64)	0.89
	PTSD-PRS	7.27 (1.07–49.66)	0.04
	Age: 40–64	0.62 (0.22–1.75)	0.36
	Age: 65–90	1.27 (0.21–7.73)	0.79
	Sex: female	1.08 (0.41–2.85)	0.88
	Psychiatric history	5.84 (2.08–16.37)	<0.001
	Prior TBI	2.19 (0.77–6.21)	0.14
	Injury cause: violence	0.52 (0.11–2.21)	0.45

*Model is adjusted for the first five ancestral principal components.
AUC = 0.779 (95% CI 0.670–0.888); conditional R^2 = 29.7%.

OR = odds ratio; CI = confidence interval; PRS = polygenic risk score; PTSD = post-traumatic stress disorder; MDD = major depressive disorder; PCL-5 = Post-traumatic Stress Disorder Checklist-5; PHQ-9 = Patient Health Questionnaire-9; AUC = area under the receiver operating characteristic curve; PC = principal component; TBI = traumatic brain injury.

Supplementary Figure S1. Subject selection flow diagram.

Numbers are provided separately for CENTER-TBI and TRACK-TBI studies (CENTER-TBI/ TRACK-TBI).

CENTER-TBI/TRACK-TBI core study

(4509/2697)

↓ → Died < 180 days post-injury (473/126)

Alive at 180 days

(4036/2571)

↓ → Age <17 (189/145)

Adults all severities

(3847/2426)

↓ → Not GCS 13-15 at recruitment (1058/451)

Eligible sample with GCS 13-15

(2744/1975)

↓ → 6-month outcome missing (1185/583)

6-month outcome (either PCL or PHQ scores)

(1559/1392)

↓ → Missing genotype data (374/169)

Genotype data available

(1185/1223)

↓ → Non-European reference populations (42/497)

↓ (from Non-European reference populations)

European study sample (1143/726)

African American study sample (0/188)

Supplementary Figure S2. Receiver operating characteristic (ROC) curves. ROC curves are presented for three models: 1) clinical variables (age, sex, psychiatric history, prior TBI, cause of injury), 2) polygenic risk score (PRS), and 3) combination of clinical variables and PRS for **(a)** PTSD and **(b)** depression following TBI in CENTER-TBI (*n* = 1143), for **(c)** PTSD and **(d)** depression following TBI in TRACK-TBI (EUR, *n* = 726), and for **(e)** PTSD and **(f)** depression following TBI in TRACK-TBI (AFR, *n* = 188). All models are adjusted for five first principal components.

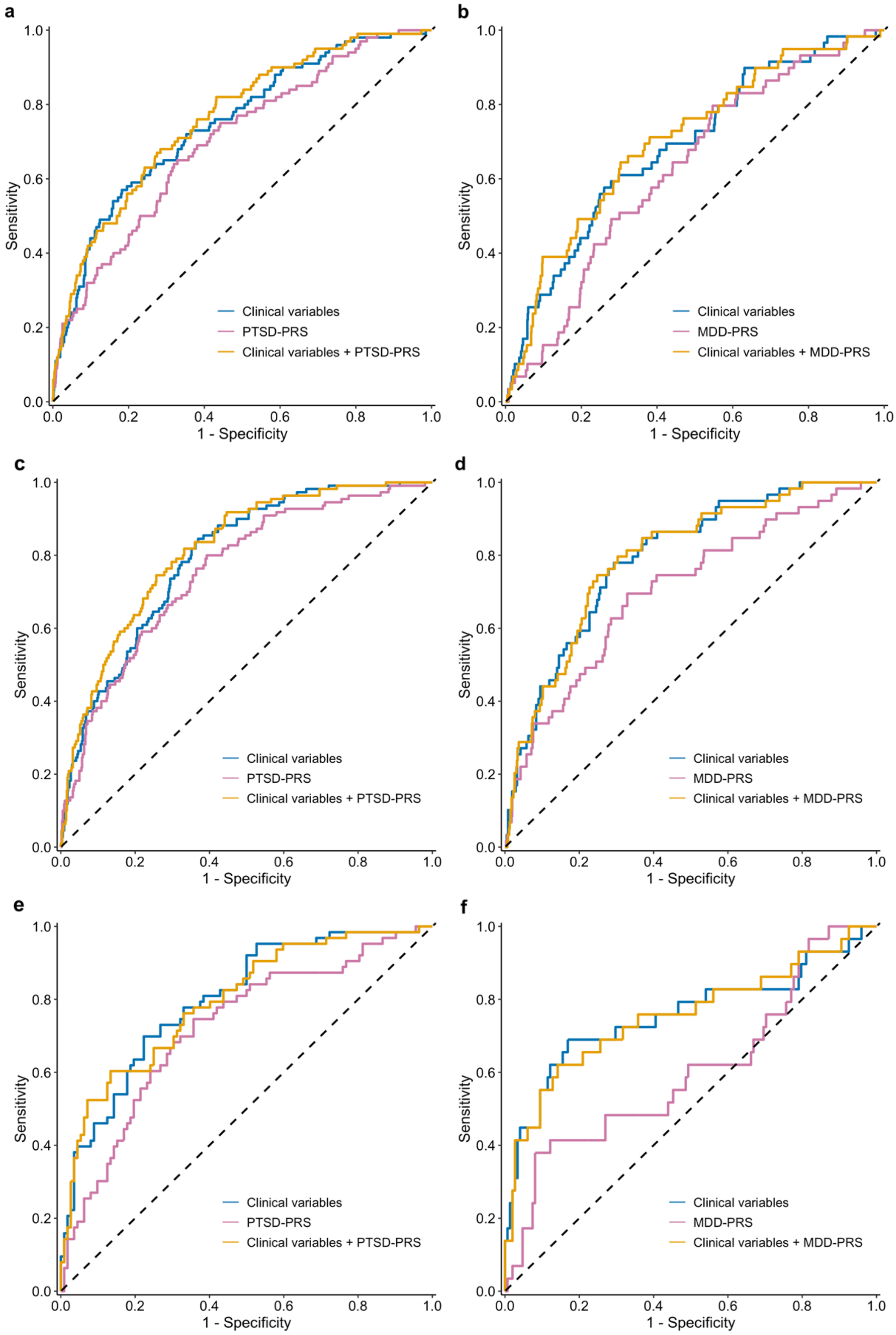

Supplementary Figure S3. Distributions of standardized polygenic risk scores (PRSs) in the TRACK-TBI cohort by reference populations. PRSs were standardized separately for European (EUR, n = 726) and African American (AFR, n = 188) individuals: **(a)** PTSD-PRS and **(b)** MDD-PRS.

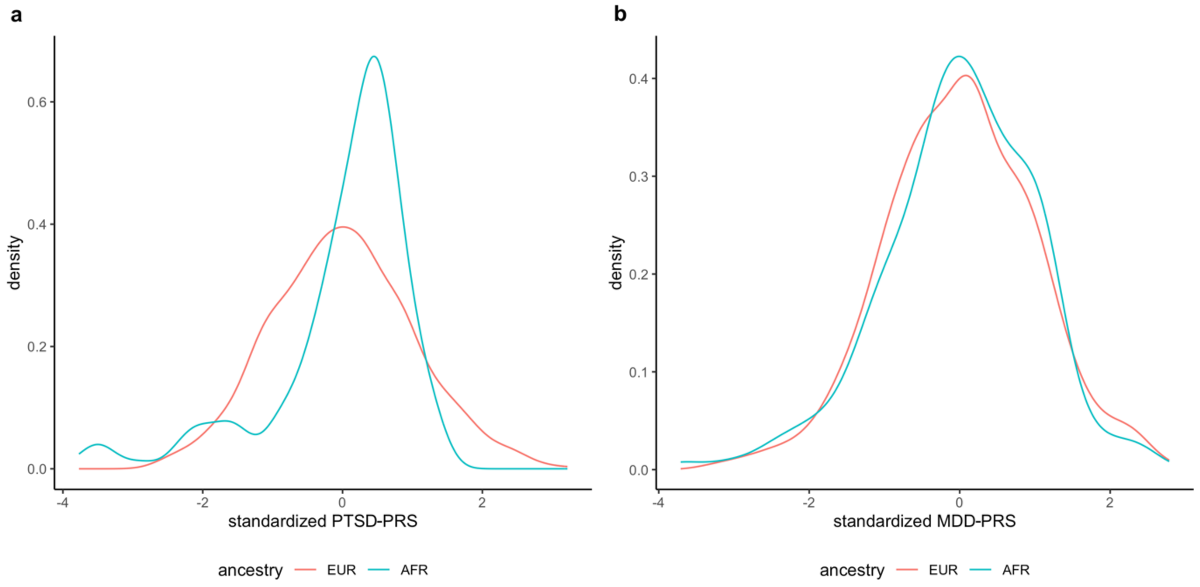

Supplement: Supplementary material [file mmc1.pdf]
